# Drought resistance index screening and evaluation of lettuce under water deficit conditions on the basis of morphological and physiological differences

**DOI:** 10.3389/fpls.2023.1228084

**Published:** 2023-09-15

**Authors:** Jingrui Li, Kumail Abbas, Lin Wang, Binbin Gong, Shenglin Hou, Weihong Wang, Bowen Dai, Hui Xia, Xiaolei Wu, Guiyun Lü, Hongbo Gao

**Affiliations:** ^1^ College of Horticulture, Hebei Agricultural University, Baoding, China; ^2^ Key Laboratory of North China Water-saving Irrigation Engineering, Hebei Agricultural University, Baoding, China; ^3^ Research Management Department, Hebei Academy of Agriculture and Forestry, Shijiazhuang, China; ^4^ College of Urban and Rural Construction, Hebei Agricultural University, Baoding, China

**Keywords:** lettuce (*Lactuca sativa* L.), water deficit, trait screening, multivariate analysis, genotype evaluation, predictive model

## Abstract

**Introduction:**

Water is one of the important factors affecting the yield of leafy vegetables. Lettuce, as a widely planted vegetable, requires frequent irrigation due to its shallow taproot and high leaf evaporation rate. Therefore, screening drought-resistant genotypes is of great significance for lettuce production.

**Methods:**

In the present study, significant variations were observed among 13 morphological and physiological traits of 42 lettuce genotypes under normal irrigation and water-deficient conditions.

**Results:**

Frequency analysis showed that soluble protein (SP) was evenly distributed across six intervals. Principal component analysis (PCA) was conducted to transform the 13 indexes into four independent comprehensive indicators with a cumulative contribution ratio of 94.83%. The stepwise regression analysis showed that root surface area (RSA), root volume (RV), belowground dry weight (BDW), soluble sugar (SS), SP, and leaf relative water content (RWC) could be used to evaluate and predict the drought resistance of lettuce genotypes. Furthermore, the drought resistance ranks of the genotypes were similar according to the drought resistance comprehensive evaluation value (D value), comprehensive drought resistance coefficient (CDC), and weight drought resistance coefficient (WDC). The cluster analysis enabled the division of the 42 genotypes into five drought resistance groups; among them, variety Yidali151 was divided into group I as a strongly drought-resistant variety, group II included 6 drought-resistant genotypes, group III included 16 moderately drought-resistant genotypes, group IV included 12 drought-sensitive genotypes, and group V included 7 highly drought-sensitive genotypes. Moreover, a representative lettuce variety was selected from each of the five groups to verify its water resistance ability under water deficit conditions. In the drought-resistant variety, it was observed that stomatal density, superoxide anion (*O*_2._^−wfi2^) production rate, and malondialdehyde (MDA) content exhibited a low increase rate, while catalase (CAT), superoxide dismutase (SOD), and that peroxidase (POD) activity exhibited a higher increase than in the drought-sensitive variety.

**Discussion:**

In summary, the identified genotypes are important because their drought-resistant traits can be used in future drought-resistant lettuce breeding programs and water-efficient cultivation.

## 1 Introduction

Lettuce (*Lactuca sativa* L.), a leafy vegetable species highly valued for its nutritional richness, holds substantial economic importance and enjoys widespread global cultivation and consumption, with China being a prominent contributor to its production (Kim et al., 2016; Shin et al., 2021). However, due to its shallow taproot system, lettuce requires frequent irrigation. Even a brief period of drought stress can adversely affect the plant, causing the production of undersized leaves and ultimately resulting in low crop yield (Li et al., 2017). To fulfill the needs of a large population, conserving water is a crucial strategy for increasing the yield of leafy vegetables, and of lettuce in particular (Saed-Moucheshi et al., 2013). Implementing such an approach can serve as an effective measure to optimize human labor in the agricultural sector while simultaneously enhancing water use efficiency (Scardigno, 2020; Hou et al., 2023).

In numerous regions worldwide, agricultural producers have prioritized the selection of crop types that can withstand drought conditions (Sun et al., 2023). Among these, drought-tolerant cultivars have demonstrated their significance in mitigating water consumption and facilitating the objective of conserving water, particularly in areas prone to high temperatures and water scarcity (Ferguson, 2019; Neha et al., 2021). Several studies have revealed that the rearing of cultivars with robust drought tolerance potential can lead to substantial reductions in water usage and notable improvements in harvest yields (Jamalluddin et al., 2021; Zhang et al., 2023). Currently, the emphasis on screening drought-tolerant varieties predominantly revolves around agronomic and field crops such as wheat (Nevo and Chen, 2010; Abdolshahi et al., 2015; Guellim et al., 2020), barley (Nevo and Chen, 2010), maize (Ali et al., 2015), cotton (Ahmad, 2020; Imtiaz et al., 2023; Iqbal et al., 2023; Wedegaertner et al., 2023), rice (Marcelo et al., 2017; Asma et al., 2021), and millet (Choudhary et al., 2021). However, there is a notable absence of comprehensive evaluations and verifications based on indicator screening for drought-tolerant genotypes specifically in lettuce. This includes assessing morphological, physiological, and biochemical changes as indicators of drought tolerance in lettuce varieties.

Over the past two decades, numerous indicators and features have been proposed to identify drought-tolerant varieties (Cai et al., 2020). Typically, drought stress indicators based on yield loss in drought conditions have been utilized for this purpose (Mohi-Ud-din et al., 2021; Sun et al., 2023). Drought susceptibility is commonly measured by comparing yield reduction under normal and water-deficient environments (Saed-Moucheshi et al., 2013). However, due to the multifaceted nature of crop genotype tolerance and susceptibility, drought tolerance in plants encompasses multiple factors, and a single indicator cannot be utilized to fully or accurately evaluate this phenotype (Munns et al., 2010; Lonbani and Arzani, 2011). Moreover, relying solely on one indicator can lead to a biased and incomplete assessment (Aslam et al., 2023). To effectively identify drought-tolerant varieties, a comprehensive evaluation of morphological, physiological, and biochemical indicators is necessary. Additionally, many evaluation indicators for assessing drought tolerance have primarily focused on filed crops, which may not be suitable for screening lettuce genotypes due to variations in growth morphology, harvested parts, and water necessities.

The identification of indicator markers for drought tolerance in genotype screening plays a critical role in various stages of plant development (Levi et al., 2009; De Brito et al., 2011; Bo et al., 2017). Previous research has identified several indicators of drought tolerance in crops. These indicators include leaf water potential, net photosynthesis, water use efficiency, maximum quantum efficiency of PSII, proline and betaine content, SS, chlorophyll, MDA, antioxidant enzyme activity, leaf area, and yield output, among others (Yan et al., 2020). However, it is acknowledged that these indicators are numerous and complex. To effectively select drought-tolerant cultivars, it is important to identify key indicators and streamline the evaluation process. In this regard, we propose the use of the CDC, D-value, and WDC to accurate assessment and screening of cultivars under both normal and drought conditions (Xu et al., 2020; Bao et al., 2023). By transforming the original set of indicators into a concise collection of representative indicators using PCA, complex and large datasets can be simplified (Donde et al., 2019). Through multiple linear regression analysis, predictive models can be developed to assess water-deficit tolerance based on morphological and physiological variables (Quevedo et al., 2022). Therefore, it is essential to establish a concise set of indicators for evaluating drought stress or develop an effective model that combines various evaluation methods to rapidly determine the resistance of superior lettuce genotypes or cultivars to drought.

Reduced yield due to water deficiency is the primary outcome of current varieties’ lack of adaptability and represents a serious danger to the ability of crops to withstand drought (Kaur and Asthir, 2017). However, plants have numerous mechanisms, such as osmotic adjustments or osmoregulation via the buildup of proline, carbohydrates, and other substances, that help them grow and produce a high yield. Plants are protected from oxidative stress by additional mechanisms, such as an increased antioxidant activity system (SOD, POD, CAT), which neutralizes ROS (OH^-^, $O_{2}{\;}_{.}^{-}$ and MDA) (Yang et al., 2021). Increased chlorophyll levels also improve photosynthesis because they allow for more efficient use of water through regulation of stomatal activity, which in turn causes a rise in CO_2_ concentration (Parida et al., 2007; Niu et al., 2018). In addition, root development is essential for sustaining water and nutrient availability during periods of drought. There is deficit of studies that employ a multivariate approach to investigate the morphological, physiological, and biochemical characteristics of lettuce genotypes and identify marker variables associated with drought resistance. Therefore, it is crucial to discover easily measurable indicators of drought stress that can facilitate rapid evaluation and validation of elite germplasm or drought-tolerant cultivars. In this experiment, 42 distinct varieties of lettuce were cultivated under both well-watered conditions (with soil water content maintained at 75%-85% of field capacity) and water-limited conditions (35%-45% of field capacity). Through the use of PCA, D-value calculation, cluster analysis, correlation analysis, and membership functional value assessment, the drought tolerance of lettuce genotypes was systematically evaluated and categorized based on their morphological and physiological indicators. Furthermore, a stepwise regression-based prediction model was developed to verify the drought tolerance capacity and mechanisms of several genotypes. These findings provide valuable insights into the drought tolerance and susceptibility of lettuce genotypes and establish a foundation for screening indicators of drought tolerance for leafy vegetables.

## 2 Materials and methods

### 2.1 Plant materials

To investigate the drought resistance of lettuce cultivars, 42 lettuce genotypes of five different types (loose leaf, butterhead, leaf heading, romaine, and mix strains) were used in this study (**Table 1**).

**Table 1:** Genotypes of 42 lettuce genotypes.

Number	Name	Cultivar groups	Number	Name	Cultivar groups
01	1902	Loose leaf	22	Jinsen	Loose leaf
02	Malvna	Loose leaf	23	Yanzhi	Loose leaf
03	Gelin	Loose leaf	24	Cuiju	Loose leaf
04	Lvdie	Butter head	25	Baoshihong	Loose leaf
05	ZiXia	Loose leaf	26	Baoshilv	Loose leaf
06	Zidie	Butter head	27	Xiangyehong	Loose leaf
07	Sheshou101	Leaf heading	28	Xiangyelv	Loose leaf
08	Hongshanhu	Loose leaf	29	Daluoma	Romaine
09	Ziya	Mix Strains	30	Luoshahong	Loose leaf
10	Lvshen	Romaine	31	Luoshalv	Loose leaf
11	Lvya	Loose leaf	32	Yidalishengcai	Loose leaf
12	Yushanhong	Mix Strains	33	Ruiluo	Romaine
13	Tehongzhou	Loose leaf	34	Naiyoushengcai	Butter head
14	Yeluo	Romaine	35	Wojulvsha	Loose leaf
15	Yidali 151	Loose leaf	36	Wojuyadan	Mix Strains
16	Jingyanyidali	Loose leaf	37	Lvbei	Leaf heading
17	Puxijin	Loose leaf	38	Meiguodasusheng	Loose leaf
18	Musi	Butter head	39	Wojunisi	Leaf heading
19	Lvluoma	Romaine	40	Lvhudie	Loose leaf
20	Ziqueshe	Loose leaf	41	Lvmeigui	Butter head
21	Kana	Mix Strains	42	Lvshanhu	Loose leaf

### 2.2 Description of a controlled environment

The experiment was carried out from October 2021 to April 2022 in Ding Xing County, Hebei Province, China. Seeds were grown in a seedling greenhouse starting on October 11, 2021, in a 105-hole tray using vermiculite:perlite:peat = 1:1:1 as substrate 25 days later and planted in a greenhouse. The physical properties of the soil are shown in **Table S1**. The average temperatures of day and night were 19.5°C and 14.0°C, respectively, and the relative humidity ranged between 45% and 55%. The temperature and relative humidity were monitored electronically by a Qingping hygrometer (Qingping Technology Co., Ltd., China). Before planting, the plots were divided into two parts: one for normal irrigation and another for water deficit irrigation. The field capacity was controlled through drip irrigation. Drip lateral lines of 16 mm diameter were laid between the two rows. The dripper discharge was 1.38 lph at a pressure of 0.1 MPa. During the whole growth period, the running time of the dripper discharge was accurately calculated according to the specified irrigation plan and the required irrigation water volume. The running time for drip irrigation was calculated as the volume of irrigation water applied divided by the number of drippers and the dripper discharge rate. The field capacity was monitored daily by the Zl6 data collector (METER Group, Inc., USA), which was inserted into the pot to a depth of 10 cm.

### 2.3 Experimental design

#### 2.3.1 Evaluation of lettuce genotypes

A factorial experiment based on a completely random design with three replications was used to evaluate lettuce genotypes under two treatments: normal irrigation (Control), in which the soil water content was maintained at 75%–85% field capacity, and water deficit (Treatment), with 35%–45% field capacity 20 days before harvest. Under the water deficit treatment, irrigation was withheld to allow the field capacity to decline to 35%–45%. At the harvest stage, 50 days after planting, 13 indexes were determined in the Horticultural College laboratory of Hebei Agricultural University, including leaf number (LN), root length (RL), root surface area (RSA), root volume (RV), average root diameter (ARD), aboveground fresh weight (AFW), belowground fresh weight (BFW), aboveground dry weight (ADW), belowground dry weight (BDW), soluble sugar content (SS), soluble protein content (SP), relative electrolytic leakage (REL), and leaf relative water content (RWC).

#### 2.3.2 Verification of lettuce with different drought resistance grades under water deficit conditions

According to the classification results of cluster analysis, one variety from each of the drought-resistant grades was selected as the material. The seedling raising method and test treatment were the same as above. At the harvest stage, the stomatal length, width, stomatal aperture, and stomatal density of the leaves; antioxidant enzyme activity including SOD, POD, and CAT activity; $O_{2}{\;}_{.}^{-}$ production rate; and MDA content were determined, with three replicates for each treatment.

### 2.4 Data collection

#### 2.4.1 Determination of morphological parameters

At the harvest stage, the morphological parameters were measured. The number of leaves was calculated manually. The plant was cut from the rhizome, and the AFW and BFW were measured. Then, the aboveground and belowground parts were dried in a ventilated oven at 105°C for 15 minutes and then dried at 75°C until a constant dry weight was achieved, and the ADW and BDW were measured.

An MRS-9600TFU2L scanner (Shanghai Zhongjing Technology Co., China) was used to scan the roots, and LA-S root analysis software (2.6.5.1) was used to analyze the root length, root surface area, root volume, and average root diameter of the plants.

#### 2.4.2 Determination of REL

The leaves were cut into long strips and placed in a calibrated tube that contained 15 ml of deionized water for 12 hours at room temperature to measure the conductivity of the extract (R1). After the leaves were heated in a boiling water bath for 30 minutes, the conductivity of the extract (R2) was measured again, and the relative conductivity was as follows: R1/R2100%. The SS was determined by the anthrone colorimetric method by (Gurrieri et al., 2020), and the SP content was determined by the coomassie brilliant blue method (Zhang et al., 2021).

#### 2.4.3 Determination of RWC

The leaves from the same part of each plant were removed at 11:00 a.m., and the fresh weight (Wf) was measured. Then, the leaves were immersed in distilled water for 5–6 hours to enable water absorption by the leaves to reach a saturation state. Afterward, the leaves were removed from the water, dried until there was no residual water on the surface, and then weighed. The saturated fresh weight (Wt) of the plant leaves was subsequently obtained. Finally, the leaves were placed in an oven, heated at 105°C for half an hour, and then dried at 85°C until a constant weight was achieved. The saturated dry weight (Wd) of the leaves and the RWC (%) = (Wf-Wd)/(Wt-Wd) 100% were subsequently obtained using the method proposed by Meher et al. (2018).

#### 2.4.4 Measurement of stomatal characteristics of the leaves

With the help of CellSens image analysis software (version 3.17.0.16686) and an Olympus BX51 fluorescence microscope (Olympus Soft Imaging Solutions GmbH), slices marked with nail polish were observed. Three slices were prepared for each variety. ImageJ image processing software was used to measure the stomatal length, stomatal width, stomatal aperture, and stomatal density in each field. After observations were made and counting was performed, the average value of the six fields was calculated (Bian et al., 2019).

#### 2.4.5 Determination of $O_{2}\overset{-}{.}$ production rate and MDA content

The method of Zhang et al. (2019) was used to determine the $O_{2}\overset{-}{.}$ production rate. The MDA content was determined according to the thiobarbituric acid method (Elstner and Heupel, 1976).

#### 2.4.6 Determination of antioxidant enzyme activity

SOD activity, POD activity, and CAT activity were assessed using a method that had been previously documented and referenced by Rahnama and Ebrahimzadeh (2004).

### 2.5 Comprehensive evaluation method

Drought resistance of different lettuce genotypes were analysed by CDC, D, and WDC according the method of Xu et al. (2020). The calculation formula is as follows:


(1) $$\text{DC=}\frac{\text{Ti}}{\text{CKi}}$$

(2) $$\;\text{CDC}=\frac{1}{\text{n}}\sum_{\text{i}=1}^{\text{n}}\text{DC}$$

(3) $$\text{ωi}=\text{Pi}÷{\;}_{\sum }^{\text{in}}\text{Pi}$$

(4) $$\text{μ}(\text{xi})=\frac{{\;}_{\text{xi}}-{\text{x}}_{\text{i},\text{min}}}{{\text{x}}_{\text{i},\text{max}}-{\text{x}}_{\text{i},\text{min}}}$$

(5) $$\text{D=}\sum_{\text{i}=1}^{\text{n}}\left[\text{μ}(\text{xi})\times \left(\text{Pi}÷{\;}_{\sum }^{\text{in}}\text{Pi}\right)\right]$$

(6) $$\text{ωi(γ)=}\gamma \text{i}÷{\;}_{\sum }^{\text{in}}\gamma \text{i}$$

(7) $$\text{WDC}=\sum_{\text{i}=1}^{\text{n}}\left[\text{DC}\times \left(\text{γi}÷\sum_{\text{i}=1}^{\text{n}}\text{γi}\right)\right]$$

According to equations (1) and (2), CDC were calculated; according to equations (3), (4) and (5), D were calculated; according to equations (6) and (7), WDC were calculated. In the formulas, T_i_ and CK_i_ represent the index measured values of the water deficit condition and normal irrigation treatment, respectively; ωi represent the factor weight coefficient; μ (xi) represent the membership function value; P_i_ is the contribution rate of the i^th^ comprehensive index, representing the importance of the ith index of all parameters, and x_i_, x_i,max_, and x_i,min_ represent the i^th^ comprehensive index, and the maximum and minimum values of the i^th^ comprehensive index respectively; The gray relational degree (γ_D_) between the DC value and D value of each parameter was subsequently obtained.

For the DC values of each index, simple correlation analysis, statistical analysis of continuous variable number distribution, and PCA were performed. When D values and DC were used as references, a stepwise regression analysis of the DC value of each index was performed to determine the corresponding regression equation. Finally, according to the D values of the lettuce genotypes evaluated, the euclidean distance and weighted pair group method average were used for cluster analysis to score the drought resistance level.

### 2.6 Data analysis

Microsoft Excel 2010 was used for data processing. PCA, stepwise regression analysis, correlations, and significance were assessed using IBM SPSS Statistics version 25 software (Armonk, NY, USA). The figures were constructed using Origin v8.0 (Origin Lab Corp., Northampton, MA, USA).

## 3 Results

### 3.1 Comprehensive evaluation of drought resistance in lettuce

#### 3.1.1 The representativeness of lettuce genotypes and analysis of measured index mean values

Drought stress had a significant impact on the measured values of various indicators of the test materials, with significant differences between treatments and materials. Under the different water treatments, 13 indicators, i.e., LN, RL, RSA, RV, ARD, AFW, BFW, ADW, BDW, SS, SP, REL, and RWC, were measured (**Table S2**). ANOVA indicated that there were significant differences among the genotypes under control and water deficit conditions (**Table S3**). The coefficient of variation (CV) between the genotypes ranged from 0.26 to 1.21 for the control and 0.07 to 1.14 for the treatment, which indicated that there is a wide range of diversity in terms of these parameters for the genotypes. The results indicated that these parameters could be suitable for use as sources for water deficit condition screening.

#### 3.1.2 Analysis of the DC in 42 lettuce genotypes

The lettuce genotypes exhibited significant variation in drought tolerance under water deficit conditions (**Table 2**). The drought coefficient (DC) for RL ranged from 0.47 to 0.97, for ADW it ranged from 0.41 to 1.00, and for SP it ranged from 0.18 to 2.12. These findings suggest the presence of lettuce genotypes with different resistance to drought. Furthermore, the coefficient of variation (CV) ranged from 0.09 to 0.50, indicating a high level of variability among the genotypes in terms of their resistance to water deficit conditions. Additionally, there were significant differences between the drought resistance coefficient of each trait for a particular genotype, highlighting the varying sensitivity of individual traits to drought stress.

**Table 2:** DC of 13 indexes in 42 lettuce genotypes.

Genotypes	LN	RL	RSA	RV	ARD	AFW	BFW	ADW	BDW	SS	SP	1/REL	RWC
1902	0.85	0.68	0.65	0.57	0.86	0.66	0.61	0.68	0.63	0.98	0.73	0.94	0.88
Malvna	0.70	0.53	0.36	0.30	0.72	0.66	0.52	0.45	0.37	0.52	0.44	0.99	0.81
Gelin	0.69	0.47	0.32	0.24	0.70	0.55	0.50	0.42	0.24	0.39	0.41	0.99	0.78
Lvdie	0.86	0.73	0.13	0.61	0.92	0.75	0.64	0.71	0.67	2.19	1.62	0.92	0.53
ZiXia	0.97	0.97	0.11	0.92	0.99	0.97	0.97	0.96	0.98	1.99	0.32	0.85	0.46
Zidie	0.95	0.93	0.92	0.88	0.98	0.94	0.91	0.92	0.91	1.69	1.86	0.78	0.98
Sheshou101	0.84	0.64	0.62	0.52	0.83	0.96	0.60	0.66	0.61	0.92	0.69	0.95	0.88
Hongshanhu	0.86	0.68	0.67	0.58	0.89	0.69	0.61	0.69	0.64	0.99	0.73	0.94	0.89
Ziya	0.67	0.47	0.19	0.02	0.43	0.67	0.39	0.41	0.20	0.31	0.36	1.00	0.74
Lvshen	0.87	0.81	0.15	0.69	0.93	0.80	0.69	0.74	0.70	1.35	1.26	0.89	0.42
Lvya	0.95	0.92	0.91	0.88	0.98	0.96	0.91	0.91	0.91	1.57	1.78	0.79	0.97
Yushanhong	0.93	0.90	0.91	0.87	0.97	0.65	0.88	0.90	0.85	1.52	1.37	0.80	0.97
Tehongzhou	0.82	0.62	0.58	0.52	0.82	0.66	0.56	0.63	0.59	0.91	0.69	0.95	0.88
Yeluo	0.92	0.86	0.88	0.80	0.96	0.92	0.84	0.87	0.81	1.35	1.20	0.83	0.96
Yidali 151	1.00	0.98	1.00	0.98	0.99	0.93	0.99	1.00	1.00	2.54	2.12	0.62	1.00
Jingyanyidali	0.92	0.84	0.87	0.77	0.96	0.90	0.84	0.84	0.78	1.30	1.08	0.85	0.95
Puxijin	0.99	0.97	0.12	0.94	0.99	0.99	0.98	0.99	0.99	0.50	0.43	0.73	0.37
Musi	0.79	0.58	0.19	0.47	0.76	0.65	0.55	0.51	0.57	1.12	1.11	0.97	0.59
Lvluoma	0.93	0.89	0.90	0.85	0.96	0.51	0.87	0.89	0.85	1.48	1.30	0.81	0.96
Ziqueshe	0.86	0.72	0.69	0.59	0.9	0.73	0.62	0.7	0.66	1.00	0.82	0.93	0.89
Kana	0.80	0.62	0.58	0.49	0.79	0.94	0.56	0.61	0.58	0.90	0.67	0.96	0.88
Jinsen	0.90	0.84	0.84	0.77	0.95	0.87	0.81	0.82	0.76	1.26	1.02	0.85	0.95
Yanzhi	0.79	0.58	0.47	0.38	0.75	0.61	0.55	0.51	0.56	0.77	0.61	0.97	0.87
Cuiju	0.92	0.85	0.88	0.79	0.96	0.92	0.84	0.85	0.78	1.35	1.11	0.84	0.96
Baoshihong	0.95	0.92	0.91	0.88	0.97	0.93	0.90	0.91	0.87	1.54	1.75	0.79	0.97
Baoshilv	0.97	0.94	0.94	0.91	0.99	0.90	0.94	0.95	0.96	1.97	2.03	0.76	0.98
Xiangyehong	0.87	0.79	0.78	0.65	0.93	0.77	0.68	0.74	0.69	1.12	0.92	0.90	0.92
Xiangyelv	0.89	0.82	0.81	0.73	0.94	0.85	0.77	0.81	0.75	1.21	0.99	0.86	0.93
Daluoma	0.91	0.84	0.84	0.77	0.95	0.45	0.83	0.82	0.76	1.27	1.07	0.85	0.95
Luoshahong	0.86	0.73	0.69	0.60	0.91	0.73	0.63	0.70	0.67	1.00	0.82	0.92	0.89
Luoshalv	0.88	0.81	0.80	0.73	0.93	0.81	0.70	0.76	0.70	1.14	0.94	0.87	0.93
Yidalishengcai	0.78	0.57	0.44	0.33	0.75	0.60	0.54	0.47	0.54	0.70	0.57	0.98	0.85
Ruiluo	0.84	0.66	0.65	0.57	0.86	1.00	0.61	0.67	0.62	0.93	0.70	0.95	0.88
Naiyoushengcai	0.89	0.82	0.83	0.74	0.94	0.86	0.78	0.81	0.75	1.23	1.02	0.85	0.94
Wojulvsha	0.72	0.53	0.44	0.32	0.74	0.57	0.53	0.47	0.50	0.58	0.56	0.98	0.82
Wojuyadan	0.92	0.87	0.11	0.84	0.96	0.92	0.86	0.88	0.82	0.57	1.75	0.83	0.62
Lvbei	0.98	0.97	0.97	0.92	0.99	0.97	0.98	0.98	0.99	2.22	2.09	0.75	1.00
Meiguodasusheng	0.87	0.79	0.75	0.63	0.92	0.76	0.68	0.72	0.68	1.06	0.90	0.91	0.91
Wojunisi	0.79	0.61	0.53	0.49	0.79	0.65	0.56	0.54	0.57	0.89	0.67	0.97	0.87
Lvhudie	0.89	0.81	0.80	0.73	0.94	0.83	0.77	0.80	0.71	1.21	0.99	0.86	0.93
Lvmeigui	0.95	0.93	0.93	0.90	0.99	0.9	0.93	0.94	0.95	1.90	1.87	0.76	0.98
Lvshanhu	0.86	0.77	0.47	0.62	0.92	0.75	0.68	0.71	0.68	1.20	0.18	0.91	0.56
AV	0.87	0.77	0.63	0.66	0.89	0.79	0.73	0.75	0.71	1.20	1.04	0.88	0.85
CV	0.09	0.19	0.44	0.33	0.13	0.19	0.22	0.23	0.26	0.42	0.50	0.10	0.20

LN, leaf number; RL, root length; RSA, root surface area; RV, root volume; ARD, average root diameter; AFW, aboveground fresh weight; BFW, belowground fresh weight; ADW, aboveground dry weight; BDW, belowground dry weight; SS, soluble sugar; SP, soluble protein; REL, relative electrolytic leakage; RWC, leaf relative water content; AV, average value; CV, coefficient of variation.

#### 3.1.3 Frequency analysis of DC of all indexes in lettuce under water deficit conditions

**Figure 1:**
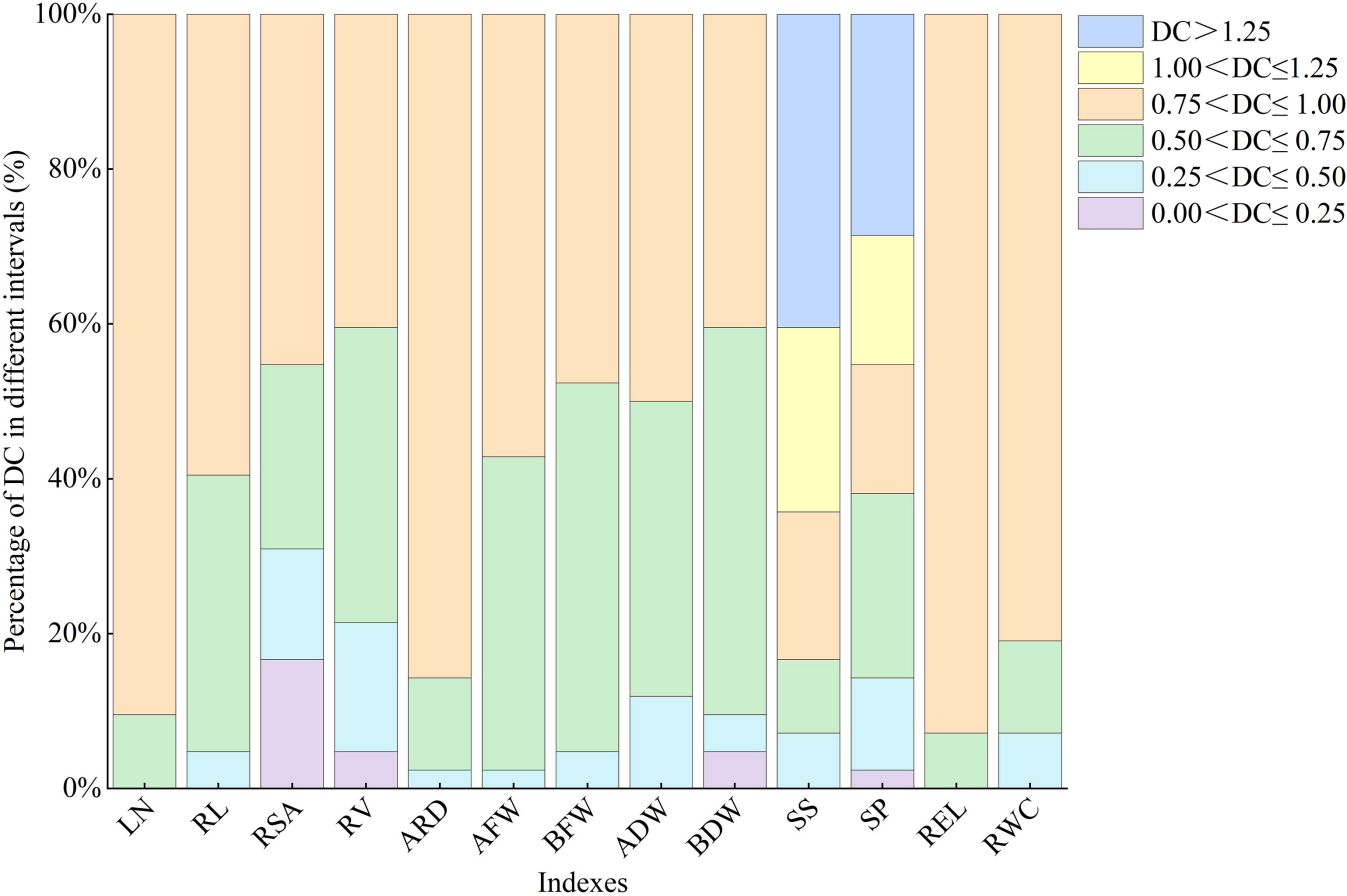
Different distributions of DC of all indexes in lettuce. LN, leaf number; RL, root length; RSA, root surface area; RV, root volume; ARD, average root diameter; AFW, aboveground fresh weight; BFW, belowground fresh weight; ADW, aboveground dry weight; BDW, belowground dry weight; SS, soluble sugar; SP, soluble protein; REL, relative electrolytic leakage; RWC, leaf relative water content.

There is significant variation in the distribution times and frequencies of DC values for each index within the same interval (**Figure 1**). SP shows a uniform distribution across six intervals, while RSA, RV, and BDW among the morphological indexes exhibit distribution across four intervals ranging from 0.0 to 1.0, indicating their high sensitivity to water deficit conditions. The DC values of LN and REL are concentrated at 0.75<DC ≤ 1, with contribution rates of 90.5% and 95.2%, respectively.

#### 3.1.4 Correlation analysis of DC of all indexes in lettuce

**Figure 2:**
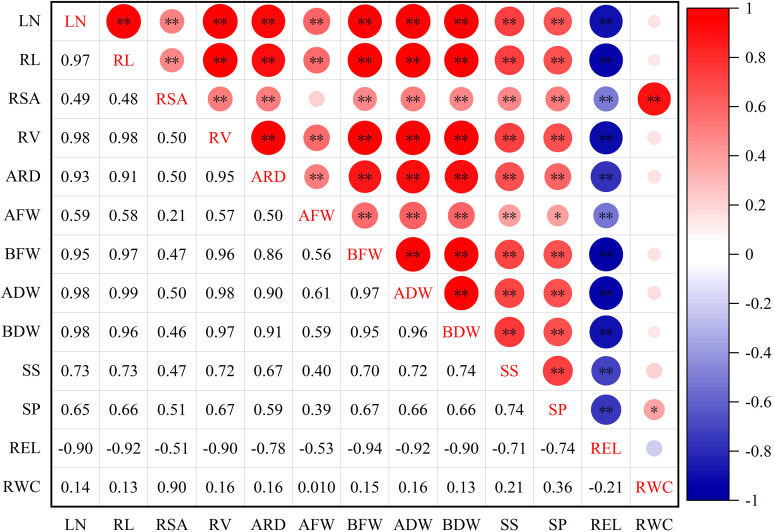
Correlation of drought resistance coefficient of each index of leaf lettuce genotypes. LN, leaf number; RL, root length; RSA, root surface area; RV, root volume; ARD, average root diameter; AFW, aboveground fresh weight; BFW, belowground fresh weight; ADW, aboveground dry weight; BDW, belowground dry weight; SS, soluble sugar; SP, soluble protein; REL, relative electrolytic leakage; RWC, leaf relative water content. *and ** indicate significant correlations at the *P*< 0.05 and *P*< 0.01 levels, respectively.

To examine the relationships between different indexes in lettuce genotypes, a correlation analysis was conducted (**Figure 2**). The results of the analysis revealed significant and high correlations among the traits. Specifically, REL exhibited significant positive correlations with LN, AFW, ADW, BFW, RC, SP, SS, ARD, and RWC. Furthermore, RWC displayed a significant correlation with SP, and highly significant correlation with RSA, while RWC had no significant correlation with other indexes.

#### 3.1.5 PCA of all indexes in lettuce genotypes

The parameters with similar effects were grouped together into distinct categories (**Table 3**). These original parameters were subsequently renamed as new independent comprehensive parameters, namely, F1, F2, F3, and F4. Among these parameters, F1 had the highest variation, primarily influenced by RV and ADW. F2 exhibited a stronger factor loading on RWC, while F3 showed a higher factor loading on AFW. In the case of F4, SP accounted for the most significant variability. The cumulative contribution of the top four factors, based on eigenvalues greater than >0.58, amounted to 94.83%.

**Table 3:** Eigenvectors and contribution rates of all indexes in lettuce genotypes by PCA.

Indexes		Component matrix			
		F1	F2	F3	F4
LN		0.977	-0.113	0.026	-0.105
RL		0.975	-0.131	-0.002	-0.106
RSA		0.584	0.778	0.144	-0.113
RV		0.980	-0.107	0.003	-0.124
ARD		0.912	-0.086	0.009	-0.224
AFW		0.609	-0.252	0.614	0.429
BFW		0.963	-0.112	-0.009	-0.087
ADW		0.980	-0.109	0.049	-0.086
BDW		0.970	-0.134	-0.012	-0.077
SS		0.792	0.079	-0.348	0.297
SP		0.755	0.236	-0.308	0.430
REL		-0.939	0.023	0.056	-0.026
RWC		0.261	0.946	0.157	-0.017
	Characteristics root	9.39	1.72	0.65	0.58
	Contribution rate (%)	72.20	13.21	4.96	4.46
	Cumulative contribution rate (%)	72.20	85.40	90.36	94.83
	Factor weights	0.76	0.14	0.05	0.05

LN, leaf number; RL, root length; RSA, root surface area; RV, root volume; ARD, average root diameter; AFW, aboveground fresh weight; BFW, belowground fresh weight; ADW, aboveground dry weight; BDW, belowground dry weight; SS, soluble sugar; SP, soluble protein; REL, relative electrolytic leakage; RWC, leaf relative water content.

#### 3.1.6 Evaluation of comprehensive drought resistance of 42 lettuce genotypes

The CDC and WDC drought resistance indexes for all lettuce genotypes, ranging from 0.45 to 1.24 and 0.59 to 1.63, respectively (**Table 4**). The mean values for CDC and WDC were 0.85 and 1.13, respectively, with corresponding CVs of 0.21 and 0.22. Based on the rankings derived from the CDC and WDC values, consistent results were obtained for the drought resistance of the 42 lettuce genotypes. Yidali 151 demonstrated strong drought tolerance, while Ziya, Malvana, and Gelin exhibited low drought resistance. The remaining genotypes showed moderate resistance to drought. Additionally, the D values for the genotypes ranged from 0.14 to 0.98, with a mean of 0.56 and a CV of 0.34. Yidali 151 displayed the highest drought resistance, while Ziya, Malvana, Wojulvsha, and Gelin were considered drought-susceptible. Other genotypes fell between these levels, aligning with the CDC and WDC values.

**Table 4:** CDC, D value and WDC of drought resistance evaluation of lettuce genotypes.

Genotypes		Comprehensive indexes				Membership function value				CDC	Sorting	D value	Sorting	WDC	Sorting
		F1	F2	F3	F4	μ1	μ2	μ3	μ4						
1902		-1.43	-0.13	-0.18	-0.19	0.47	0.36	0.63	0.63	0.76	30	0.47	30	1.00	30
Malvna		-4.89	-0.18	0.38	0.39	0.19	0.34	0.78	0.78	0.57	40	0.27	40	0.74	40
Gelin		-5.81	-0.23	0.71	0.74	0.12	0.33	0.87	0.87	0.52	41	0.22	41	0.68	41
Lvdie		-0.75	-1.09	-0.37	-0.39	0.52	0.13	0.58	0.58	0.88	19	0.47	29	1.14	21
Zixia		2.15	-1.11	-2.54	-2.67	0.75	0.12	0.00	0.00	0.91	15	0.59	21	1.21	15
Zidie		3.58	0.5	0.53	0.56	0.86	0.50	0.82	0.82	1.09	5	0.81	5	1.45	5
Sheshou101		-1.27	-0.26	-0.15	-0.16	0.48	0.32	0.64	0.64	0.76	31	0.47	28	1.00	31
Hongshanhu		-1.19	-0.17	-0.21	-0.23	0.49	0.35	0.62	0.62	0.77	29	0.48	27	1.01	29
Ziya		-7.29	0	0.71	0.74	0.00	0.39	0.87	0.87	0.45	42	0.14	42	0.59	42
Lvshen		-0.75	-1.64	-0.24	-0.26	0.52	0.00	0.62	0.61	0.81	25	0.46	31	1.07	25
Lvya		3.48	0.39	0.52	0.55	0.86	0.48	0.82	0.82	1.07	6	0.80	6	1.43	6
Yushanhong		2.3	0.68	0.11	0.12	0.76	0.55	0.71	0.71	1.00	8	0.73	8	1.33	8
Tehongzhou		-2.25	-0.06	-0.09	-0.09	0.40	0.37	0.66	0.66	0.72	34	0.42	34	0.94	34
Yeluo		2.23	0.32	0.03	0.03	0.76	0.46	0.69	0.69	0.97	10	0.71	9	1.30	10
Yidali 151		5.28	2.61	0.48	0.51	1.00	1.00	0.81	0.81	1.24	1	0.98	1	1.63	1
Jingyanyidali		1.85	0.21	-0.02	-0.02	0.73	0.44	0.68	0.67	0.94	12	0.68	12	1.26	12
Puxijin		1.75	-0.85	-0.93	-0.97	0.72	0.19	0.43	0.43	0.82	24	0.62	19	1.12	22
Musi		-3.49	-1.03	0.1	0.1	0.30	0.14	0.71	0.70	0.69	36	0.32	38	0.88	36
Lvluoma		1.85	0.7	0.04	0.04	0.73	0.55	0.69	0.69	0.97	9	0.70	10	1.30	9
Ziqueshe		-0.8	-0.15	-0.07	-0.08	0.52	0.35	0.66	0.66	0.79	27	0.51	25	1.04	27
Kana		-1.92	-0.21	-0.07	-0.08	0.43	0.34	0.66	0.66	0.73	33	0.44	32	0.96	33
Jinsen		1.47	0.21	-0.03	-0.03	0.70	0.44	0.67	0.67	0.92	13	0.66	13	1.23	13
Yanzhi		-3.51	0.01	0	0	0.30	0.39	0.68	0.68	0.65	37	0.35	36	0.84	37
Cuiju		2.05	0.25	-0.02	-0.02	0.74	0.44	0.68	0.67	0.95	11	0.69	11	1.28	11
Baoshihong		3.24	0.37	0.57	0.6	0.84	0.47	0.83	0.83	1.06	7	0.79	7	1.41	7
Baoshilv		4.07	0.77	0.46	0.49	0.90	0.57	0.80	0.80	1.14	3	0.85	3	1.51	3
Xiangyehong		0.06	-0.01	-0.03	-0.03	0.58	0.38	0.67	0.67	0.84	22	0.57	22	1.12	23
Xiangyelv		1.08	0.18	-0.07	-0.07	0.67	0.43	0.66	0.66	0.90	16	0.63	15	1.20	16
Daluoma		0.7	0.46	0.02	0.03	0.64	0.49	0.69	0.69	0.89	17	0.62	17	1.19	17
Luoshahong		-0.68	-0.13	-0.08	-0.09	0.53	0.36	0.66	0.66	0.80	26	0.52	24	1.05	26
Luoshalv		0.52	0.18	0.02	0.03	0.62	0.43	0.69	0.69	0.87	21	0.60	20	1.15	20
Yidalishengcai		-3.88	-0.06	0.07	0.07	0.27	0.37	0.70	0.70	0.63	38	0.33	37	0.80	38
Ruiluo		-0.88	-0.34	-0.16	-0.17	0.51	0.31	0.64	0.64	0.77	28	0.49	26	1.02	28
Naiyoushengcai		1.23	0.19	-0.04	-0.04	0.68	0.43	0.67	0.67	0.91	14	0.64	14	1.21	14
Wojulvsha		-4.45	-0.11	0.17	0.18	0.23	0.36	0.73	0.73	0.60	39	0.30	39	0.77	39
Wojuyadan		0.94	-1.09	1.19	1.26	0.65	0.13	1.00	1.00	0.87	20	0.62	18	1.18	19
Lvbei		4.74	0.91	0.3	0.32	0.96	0.60	0.76	0.76	1.18	2	0.89	2	1.57	2
Meiguodasusheng		-0.12	-0.07	0	0	0.57	0.37	0.68	0.68	0.83	23	0.55	23	1.10	24
Wojunisi		-2.82	-0.09	-0.03	-0.04	0.36	0.36	0.67	0.67	0.69	35	0.39	35	0.90	35
Lvhudie		0.92	0.18	0.02	0.02	0.65	0.43	0.69	0.68	0.89	18	0.62	16	1.19	18
Lvmeigui		3.82	0.76	0.32	0.34	0.88	0.56	0.77	0.77	1.11	4	0.83	4	1.48	4
Lvshanhu		-1.15	-0.92	-1.43	-1.5	0.49	0.17	0.30	0.30	0.73	32	0.42	33	0.97	32
	Average value	–	–	–	–	–	–	–	–	0.85	–	0.56	–	1.13	–
	Coefficient of variation	–	–	–	–	–	–	–	–	0.21	–	0.34	–	0.22	–

F1, F2, F3 and F4 represent the comprehensive indexes, and μ1, μ2, μ3 and μ4 represent the subordinate function values of the five factors. CDC, comprehensive drought resistance coefficient; D value, drought resistance comprehensive evaluation value; WDC, weight drought resistance coefficient.

#### 3.1.7 Analysis of the gray relational degree for all studied traits under water deficit conditions

The correlation between the DC and D values for each studied trait was ranked in ascending order as follows: BDW, RSA, RV, BFW, ADW, RWC, SS, SP, AFW, LN, ARD, and REL, with BDW being the most strongly correlated trait. This ranking primarily reflects the similarity between the DC and D values for each index, which aligns with the susceptibility of each variety to water deficit conditions (**Table 5**). Furthermore, the correlation between the DC and WDC of each index was also ranked, with belowground dry weight exhibiting the most significant correlation among all the traits considered in relation to the correlation between DC and CDC.

**Table 5:** Correlation degree between DC and CDC, DC and D value, DC and WDC of each index and the weight of each index in lettuce genotypes.

Indexes	DC and CDC			DC and D value			DC and WDC		
	Correlation degree	Sorting	Weight coefficient	Correlation degree	Sorting	Weight coefficient	Correlation degree	Sorting	Weight coefficient
LN	0.83	7	0.08	0.91	10	0.08	0.91	10	0.08
RL	0.69	12	0.07	0.55	13	0.05	0.55	13	0.05
RSA	0.90	2	0.08	0.96	2	0.08	0.97	2	0.08
RV	0.82	9	0.08	0.96	3	0.08	0.96	3	0.08
ARD	0.84	5	0.08	0.90	11	0.08	0.90	11	0.08
AFW	0.82	8	0.08	0.92	9	0.08	0.93	9	0.08
BFW	0.89	4	0.08	0.95	4	0.08	0.96	4	0.08
ADW	0.90	3	0.09	0.95	5	0.08	0.95	5	0.08
BDW	0.91	1	0.09	0.96	1	0.08	0.97	1	0.08
SS	0.76	10	0.07	0.95	7	0.08	0.95	6	0.08
SP	0.67	13	0.06	0.94	8	0.08	0.95	8	0.08
REL	0.75	11	0.07	0.79	12	0.07	0.79	12	0.07
RWC	0.84	6	0.008	0.95	6	0.08	0.95	7	0.08

LN, leaf number; RL, root length; RSA, root surface area; RV, root volume; ARD, average root diameter; AFW, aboveground fresh weight; BFW, belowground fresh weight; ADW, aboveground dry weight; BDW, belowground dry weight; SS, soluble sugar; SP, soluble protein; REL, relative electrolytic leakage; RWC, leaf relative water content. DC, drought resistance coefficient; CDC, comprehensive drought resistance coefficient; D value, drought resistance comprehensive evaluation value; WDC, weight drought resistance coefficient.

#### 3.1.8 Screening of drought resistance indicators by stepwise regression analysis

The measured DC values were subjected to regression analysis together with the D value, and the coefficient of determination (R^2^) was 0.985. The F test value was highly significant, which indicated that the regression equation was optimal, that the predictions were accurate and that the model was a good fit for the given data (**Table 6**). According to the regression equation between the D value and DC value of each index, the drought resistance of lettuce genotypes can be identified by measuring the indexes closely related to the D value, such as RSA, RWC, RV, BDW, SS, and SP, thus simplifying the identification work.

**Table 6:** Drought resistance model prediction in lettuce genotypes by stepwise regression analysis.

Multiple regressive equations	Coefficient of determination R^2^	F-value	P-value		Correlation coefficient R	
				CDC	D value	WDC
y= -0.399 + 0.520x_3_ + 0.302x13 + 0.181x_4_ + 0.151x9 + 0.058x11 + 0.016x_10_	0.985	455.41	<0.01	0.986**	1	0.991**

X_3_: root surface area; X_4_: root volume; X_9_: belowground dry weight; X_10_: soluble sugar; X_11_: soluble protein; X_13_: leaf relative water content. ** indicates a significant difference at P< 0.01.

#### 3.1.9 Cluster analysis and classification of drought resistance levels

**Figure 3:**
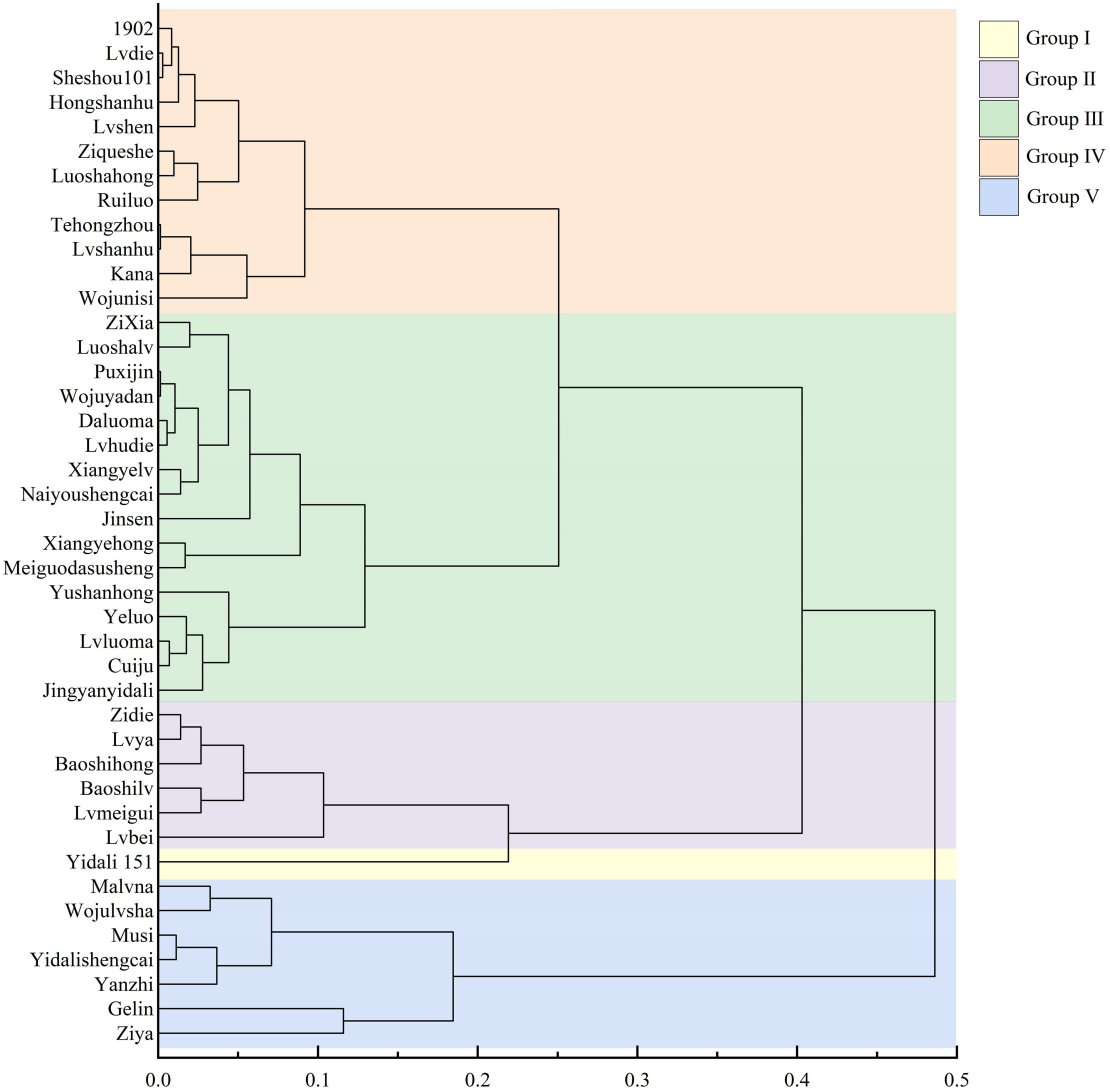
Cluster diagram of drought resistance in lettuce cultivars based on D value. Group I, Group II, Group III, Group IV, and Group V represent different drought resistance levels.

Using cluster analysis with a threshold value (λ) set at 5, the 42 lettuce genotypes were classified into five distinct groups based on their D values (**Figure 3**). Group I consisted of drought-resistant genotypes, with Yidali 151 being the most prominent among themand exhibiting the highest D value (**Table S4**). Group II, Group III, Group IV, and Group V included 6 drought-resistant genotypes, 16 moderately drought-resistant genotypes, 12 drought-sensitive genotypes, and 7 highly drought-sensitive genotypes, respectively. The proportions of genotypes in groups I, II, III, IV, and V were 2.3%, 14.3%, 38.1%, 28.6%, and 16.7%, respectively, encompassing the entire set of evaluated genotypes.

### 3.2 Verification of the comprehensive analysis and evaluation system for the water deficit adaptability of lettuce

#### 3.2.1 Morphological changes in five lettuce genotypes under water deficit conditions

**Figure 4:**
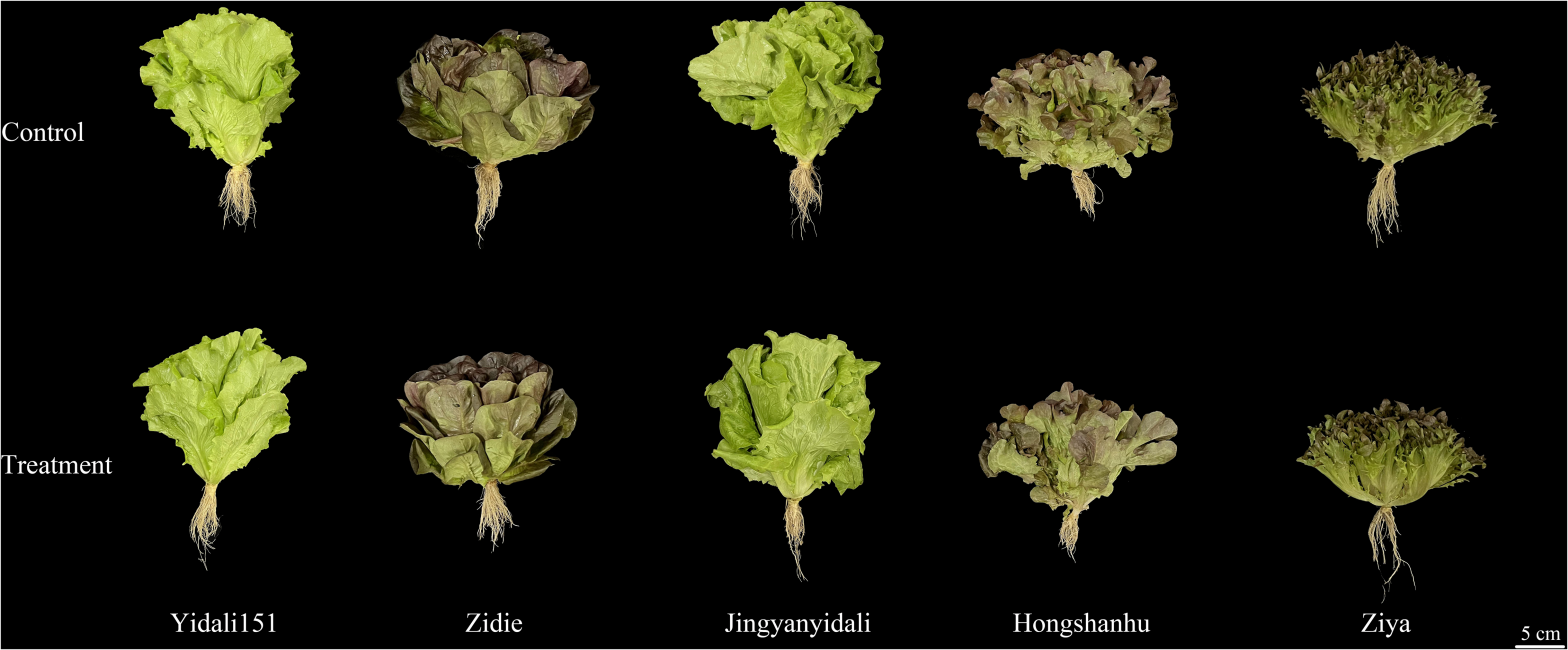
Morphological changes in five lettuce genotypes under water deficit for both experimental groups.

To validate the accuracy of the experimental model, five lettuce genotypes representing different levels of drought resistance were selected: Yidali151, Zidie, Jingyanyidali, Hongshanhu, and Ziya. When subjected to water-deficit conditions, these five genotypes displayed noticeable variations and significant differences among them (**Figure 4**).

#### 3.2.2 Stomatal traits of the upper epidermis of the leaves

**Figure 5:**
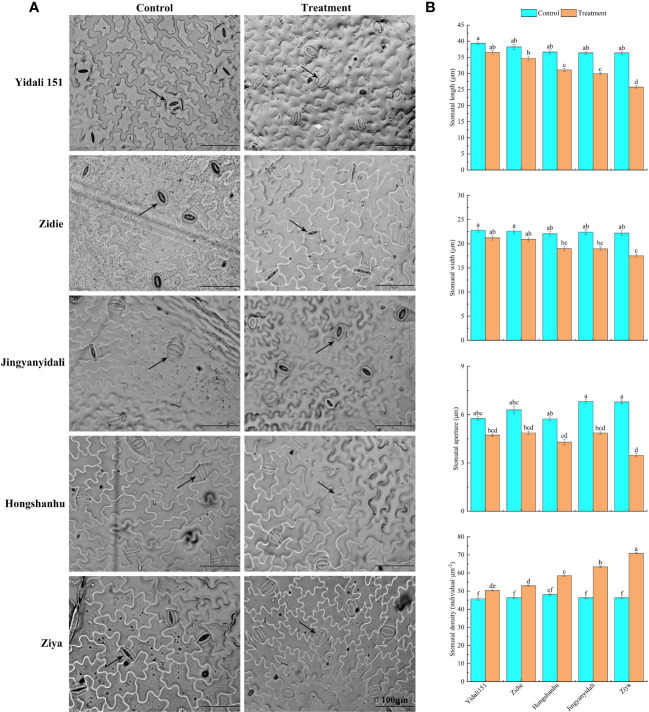
Stomatal traits in the upper epidermis of lettuce under water deficit conditions. **(A)** Differences in stomatal traits in the upper epidermis of five genotypes under normal irrigation and water deficit conditions. **(B)** Stomatal length, width, stomatal aperture and stomatal density in the upper epidermis of five genotypes under normal irrigation and water deficit treatments. Different letters indicate significant differences among various genotypes and treatments according to LSD (*p* ≤ 0.05), and vertical bars represent standard errors.

The upper epidermal stomatal traits exhibited variations among different lettuce genotypes under water-deficit conditions (**Figure 5A**). In comparison to the control group, the stomatal length, width, and aperture decreased in the five lettuce genotypes subjected to water deficit conditions. Specifically, the stomatal length and width experienced reductions ranging from 7.3% to 29.1% and 6.8% to 21.2%, respectively. Moreover, the stomatal aperture significantly decreased, ranging from 18.0% to 48.8%, across the different genotypes. Interestingly, under water deficit conditions, the stomatal densities of Yidali151, Zidie, Jingyanyidali, Hongshanhu, and Ziya increased by 8.8%, 14.3%, 21.5%, 36.8%, and 55.2%, respectively (**Figure 5B**).

#### 3.2.3 Stomatal traits of lower epidermis of the leaves

**Figure 6:**
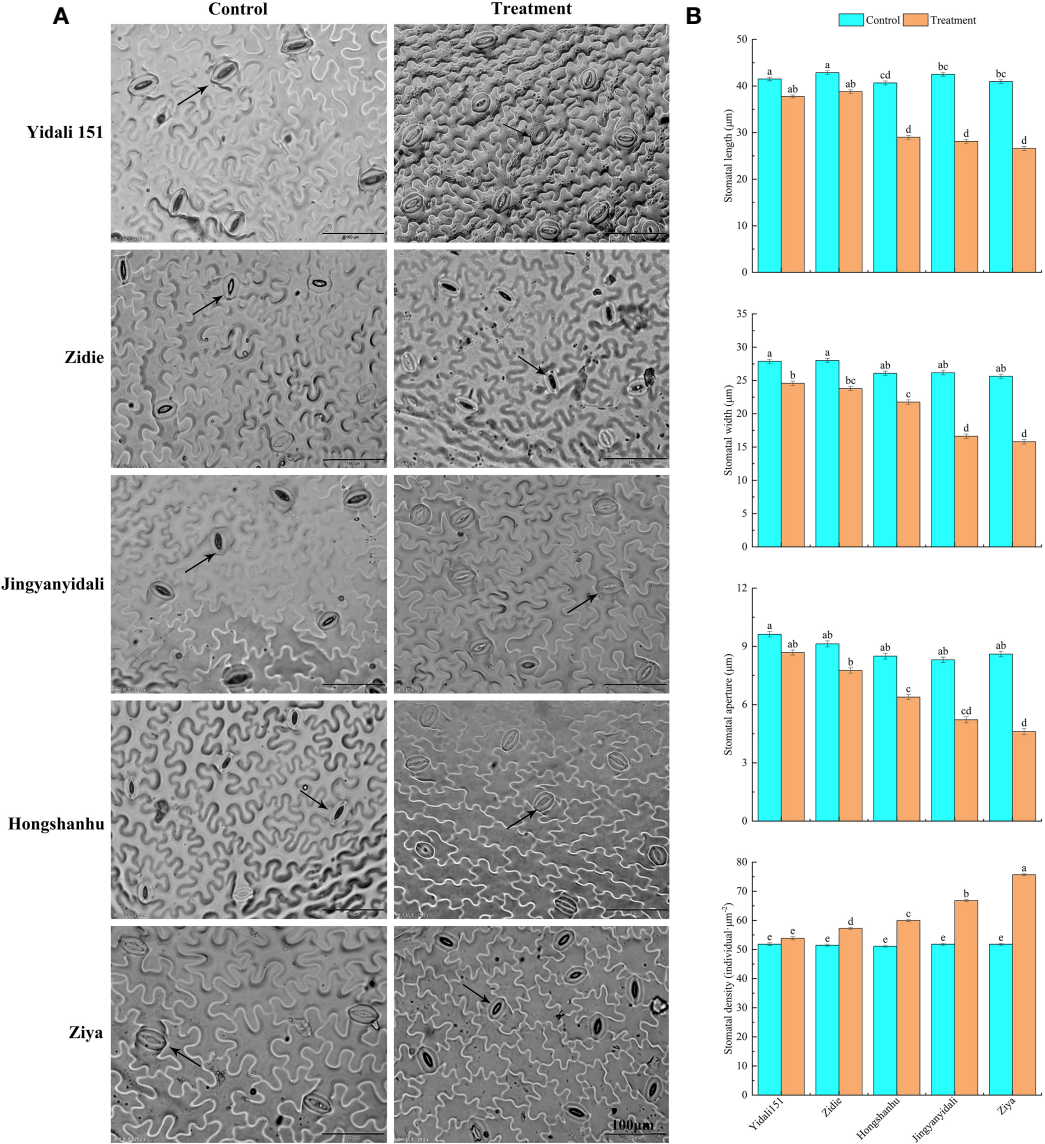
Differences in stomatal traits in the lower epidermis of five genotypes under water deficit conditions. **(A)** Differences in stomatal traits in the lower epidermis of five genotypes under normal-irrigation and water deficit conditions. **(B)** Stomatal length, width, stomatal aperture and stomatal density in the lower epidermis of five genotypes under normal irrigation and water deficit treatments. Different letters indicate significant differences among various genotypes and treatments according to LSD (*p* ≤ 0.05), and vertical bars represent standard errors.

Under water-deficit conditions, the lower epidermal stomatal traits displayed variations among the five lettuce genotypes (**Figure 6A**). The stomatal length of Yidali151, Zidie, and Jingyanyidali did not show significant differences between normal irrigation and water deficit conditions. However, Hongshanhu and Ziya exhibited a significant decrease in stomatal length. The stomatal width of all five lettuce genotypes significantly decreased by 11.8% to 38.4% under water-deficit conditions. Similarly, the stomatal aperture decreased in all genotypes, with Ziya showing a significant decrease. In terms of stomatal density, Yidali151 demonstrated the lowest difference (3.9%) between normal irrigation and water deficit conditions, while Ziya exhibited the highest difference (46.1%) (**Figure 6B**).

#### 3.2.4 $O_{2}\overset{-}{.}$ Production rate and MDA content of lettuce under water deficit conditions

**Figure 7:**
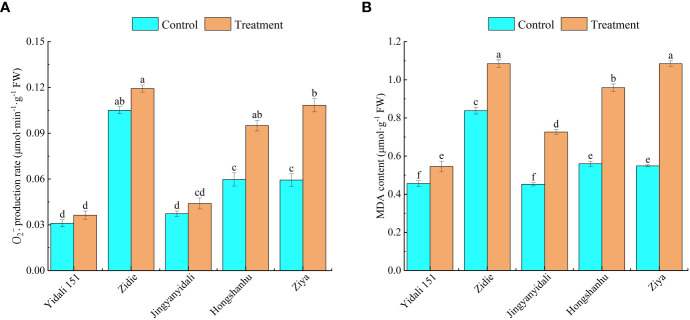
$O_{2}\bar{.}$ production rate **(A)** and MDA content **(B)** of lettuce under water deficit conditions. Different letters indicate significant differences among various genotypes and treatments according to LSD (P≤0.05), and vertical bars represent standard errors.

Under water deficit conditions, the production rate of $O_{2}\overset{-}{.}$ and MDA content showed variations among different genotypes. The $O_{2}\overset{-}{.}$ production rates of Yidali151, Zidie, and Jingyanyidali did not exhibit significant differences between normal irrigation and water deficit conditions. However, Hongshanhu and Ziya experienced increases of 59.1% and 82.6%, respectively, in their $O_{2}\overset{-}{.}$ production rates (**Figure 7A**). Furthermore, compared to the control group, the MDA contents significantly increased under water deficit conditions. Yidali151, Zidie, Jingyanyidali, Hongshanhu, and Ziya demonstrated increases of 19.1%, 29.5%, 60.8%, 71.2%, and 97.8%, respectively, in their MDA contents (**Figure 7B**).

#### 3.2.5 Antioxidant enzyme activity of lettuce under water deficit conditions

**Figure 8:**
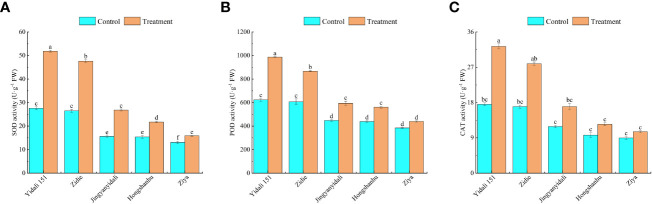
SOD **(A)**, POD **(B)**, CAT **(C)** activity of lettuce under water deficit conditions. Different letters indicate significant differences among various genotypes and treatments according to LSD (p≤0.05), and vertical bars represent standard errors.

Under water deficit conditions, the activities of SOD, POD, and CAT in the five genotypes increased (**Figure 8**). Specifically, SOD activity significantly increased in all genotypes, with Yidali151 exhibiting the highest increase of 88.1% and Ziya showing the lowest increase of 21.5%. In comparison to the control group, the POD activity in Yidali151, Zidie, Jingyanyidali, and Hongshanhu increased by 58.4%, 42.9%, 32.6%, and 28.0%, respectively, under water deficit conditions. However, no significant difference was observed in POD activity for Ziya between normal irrigation and water deficit conditions. Regarding CAT activity, Yidali151, Zidie, and Jingyanyidali demonstrated significant increases of 84.2%, 64.5%, and 42.6%, respectively, under water deficit conditions. On the other hand, no significant difference in CAT activity was observed for Hongshanhu and Ziya between normal irrigation and water deficit conditions.

## 4 Discussion

Drought stress significantly affects plant morphology, physiology, and yield, leading to reduced overall performance (Claeys and Inzé, 2013). Previous studies have often focused on specific aspects such as morphology, photosynthesis, or physiology to assess drought tolerance. However, it is crucial to consider indicators related to both yield and morphological characteristics to comprehensively evaluate drought tolerance (Dolferus, 2014; Ndayiragije and Li, 2022). In evaluating drought tolerance in cotton, morphological and yield indicators have been commonly used (Sun et al., 2021). However, these indicators provide only a partial understanding of crop drought tolerance, neglecting the roles of photosynthesis, physiology, and biochemical attributes. Osmotic adjustment, including parameters such as MDA and proline content, is an important component of drought tolerance and should be incorporated into comprehensive assessments (Wei et al., 2009; Fang and Xiong, 2015). Therefore, when evaluating and verifying drought tolerance, it is necessary to integrate multiple indicators that encompass morphology, physiology, biochemical changes, and representative indexes. This comprehensive approach ensures a better understanding of drought tolerance screening and evaluation in plants.

In our study, we conducted field cultivation of 42 lettuce genotypes and evaluated 13 indicator variables associated with drought tolerance. These indicators encompassed morphological, physiological, and biochemical traits. Throughout the growth stages, ranging from seedling to harvest, we performed variance analysis on the drought tolerance coefficient for the different water treatments. Notably, significant differences were observed among the genotypes, particularly under drought stress conditions (**Table 2**). These findings demonstrate the presence of substantial genetic diversity among the genotypes, making them representative samples for the region. Notably, drought stress had a significant impact on all the indicators examined (*P*<0.05), leading to decreased drought tolerance coefficients (<1) or increased drought tolerance coefficients (>1) (**Table 2**). Furthermore, the CV values for most indicators were higher under water deficit conditions compared to the normal treatment. This observation suggests that the lettuce genotypes chosen for this study exhibit considerable diversity, effectively responding to drought stress and generating representative outcomes.

To account for inherent variations among cultivars, we utilized relative values in evaluating different pakchoi varieties under drought stress. However, drought tolerance is a complex trait influenced by multiple factors, and relying on individual or single-type indicators for assessment can lead to errors (Ndayiragije and Li, 2022; Xiong et al., 2022). Currently, there is no single indicator that can provide a complete and accurate evaluation of drought tolerance. Therefore, it is crucial to identify more comprehensive indicators and adopt suitable evaluation methods for plant assessments. Moreover, many indicators exhibit correlations with each other, resulting in overlapping responses as indicators of crop stress tolerance (**Figure 2**). Consequently, the utilization of multivariate analysis methods becomes essential in evaluating and screening comprehensive indicators associated with drought tolerance. PCA has proven to be effective in reducing multiple variables to underlying factors, addressing missing data issues, and facilitating efficient grouping of drought-tolerant varieties (Wu and Bao, 2012; Maheswari et al., 2016). Through PCA, we successfully transformed the 13 individual indicators of lettuce genotypes under drought stress into four distinct independent comprehensive indexes. Notably, the cumulative contribution rate of the first four independent comprehensive indexes exceeded 94.83% (**Table 3**), indicating that a significant portion of the data encompassed by the 13 indicators was effectively encompassed by these comprehensive indexes. Previous studies utilized the PCA method, and the Zhong R2016 and XinLuZao 45 cultivars, two highly drought-tolerant cotton genotypes, were identified among 104 cotton genotypes based on 19 drought-related indicators (Sun et al., 2021). The drought tolerance membership function value acts as a multivariate indicator that combines the drought tolerance coefficients of different indicators, providing a comprehensive representation of overall plant performance under drought stress. This indicator effectively captures and reflects the collective response of plants to drought conditions. By utilizing the principal component scores, we calculated the membership function values and subsequently determined the D-value by incorporating the respective weights. This approach enabled us to rank the lettuce genotypes based on their drought tolerance, with higher D-values indicating greater drought tolerance (**Table 4**). Similar findings were demonstrated by (Liu et al., 2015), and the results showed that twenty-six of the 82 wheat addition lines that expressed high drought resistance were selected by membership function value based on 10 agronomic traits. A higher MFVD (membership functional value of drought resistance) was observed in the *Agropyron elongatum* 3E addition line, which was considered the most drought-resistant material. Previous studies have utilized the classification of onion cultivars based on waterlogging tolerance and wheat and maize varieties based on salt tolerance. In these studies, the cultivars or varieties were categorized into two groups using their respective characteristics. The classification was determined by evaluating the euclidean distances between the cultivars or varieties, which provided valuable insights into their comparative levels of tolerance (Huqe et al., 2021; Uzair et al., 2022). Likewise, the drought tolerance of cotton cultivars was classified using the membership function and D-value. This classification approach allowed for the grouping of cotton cultivars into distinct categories based on their individual levels of drought tolerance. This study observed significant differences in morphological, physiological, and biochemical characteristics among the lettuce genotypes, indicating the presence of abundant genetic diversity. PCA was employed to transform the 13 indexes of drought tolerance in lettuce varieties into 4 independent composite indicators according to previous studies. The D-values of various lettuce genotypes were then determined using the membership function. Combining CDC, D-values, and WDC analysis enhances the reliability and practicality of stress tolerance assessment in lettuce. Through stepwise regression analysis, it was determined that among the 13 studied indexes, six drought tolerance indexes (RSA, RV, BDW, SS, SP, and RWC) had significant effects on the drought tolerance of lettuce. These indexes can be considered primary indexes for the evaluation and screening of drought-tolerant lettuce genotypes in future studies. Moreover, a stepwise regression predictive model was developed to assess the drought tolerance of lettuce, represented by the equation Y= -0.399 + 0.520RSA + 0.302RWC + 0.181RV + 0.151BDW + 0.058SP + 0.016SS (R^2 ^= 0.985, *P* value< 0.01). By incorporating multiple indexes as predictors, this model offers a dependable approach for evaluating the drought tolerance of lettuce. Through hierarchical clustering analysis, the 42 lettuce genotypes were classified into 5 diverse categories based on their D-values: highly drought- resistant, moderately drought- resistant, drought- resistant, drought-sensitive, and highly drought-sensitive (**Figure 3**). The results of the gray relational analysis further validate the accuracy of the regression analysis and enhance the scientific reliability and credibility of the identified indexes in assessing drought tolerance.

Comprehensive evaluation methods have proven highly effective in identifying drought-tolerant plant varieties. The evaluation process encompasses various morphological and physiological aspects that contribute to a plant’s ability to withstand drought. It is crucial to validate the classification of plants using comprehensive evaluation methods that consider multiple indicators. One of the key indicators for drought tolerance is stomatal behavior, including their patterning and morphology, which significantly influence water use efficiency. Stomata, found on the surfaces of leaves, exhibit a wide range of shapes, sizes, and numbers across different plant species (Liu et al., 2021). The variations in stomatal size and density can be attributed to genetic factors as well as plant growth under diverse environmental conditions. When subjected to drought stress, stomata close to reduce water loss through evapotranspiration, resulting in decreased stomatal density on both the upper and lower epidermis. Our results demonstrated that with an increase in drought stress, the stomata close, and their density in the upper and lower epidermis also decreases to prevent evapotranspiration. Similar results were observed in wheat crop cultivars that showed lower stomatal density under well-watered and water-stressed conditions, and the drought-sensitive cultivar had a nonsignificantly larger decrease under water-stressed conditions (Nyachiro et al., 2001). In the current study, the significant changes in stomatal traits of drought-resistant genotypes helped to improve drought resistance, such as the reduction in stomatal aperture and density under water deficit conditions. The stomatal aperture of drought-sensitive lettuce decreased more than that of drought-resistant genotypes, and the stomatal density of drought-sensitive lettuce increased more than that of drought-resistant genotypes (**Figure 6**).

ROS play a crucial role in the metabolic pathways associated with plant drought tolerance. Under drought stress conditions, plants frequently exhibit heightened levels of ROS, including increased production of $O_{2}\bar{.}$ and elevated H_2_O_2_ content. H_2_O_2_ production intensifies at the onset of drought stress against which CAT is synthesized. in response to which antioxidants are produced to scavenge the ROS. SOD catalyzes the dismutation of superoxide to molecular oxygen and H_2_O_2_, after which H_2_O_2_ is converted into water and oxygen inside the cytosol and chloroplasts, which protects the cell from the toxic effects of ROS (Sarker and Oba, 2018; Liu et al., 2022). The high activity of SOD, CAT, and POD inside the cell during drought stress reflects the ability of the genotypes to tolerate drought; therefore, the genotypes in which high concentrations of these antioxidants are generated were considered tolerant, and the genotypes that produced low concentrations were considered sensitive. For different drought genotypes of soybean, the antioxidant enzyme activities of SOD, CAT, and POD in drought-resistant genotypes exceeded those of sensitive genotypes under different treatment times and drought degrees (Zhou et al., 2022). Antioxidant enzymes are commonly utilized as physiological indicators to identify plant stress resistance (Sánchez-Rodríguez et al., 2010; Sallam et al., 2019). In this study, the $O_{2}\overset{-}{.}$ and MDA contents increased in five genotypes under water deficit conditions. The $O_{2}\overset{-}{.}$ production rates of Yidali151, Zidie, and Jingyanyidali showed no significant difference, and Ziya significantly increased by 82.6%, and the MDA contents of Yidali151 and Ziya increased by 19.1% and 97.8%, respectively. Under water deficit conditions, the SOD, POD and CAT activities increased 88.1%, 58.4%, and 84.2%, respectively, in Yidali151. In Ziya, SOD increased by 21.5%, and POD and CAT showed no significant difference between normal irrigation and water-deficit conditions.

Therefore, in the evaluation and analysis of water deficit tolerance, it is crucial to account for a comprehensive range of indicators encompassing morphological, physiological, and biochemical parameters. This comprehensive approach enhances the effectiveness of identifying and screening drought-tolerant pakchoi varieties while reducing the cost and time associated with phenotyping. However, further research is required to unravel the molecular mechanisms underlying drought tolerance in lettuce genotypes and to develop targeted breeding strategies for highly drought-tolerant genotypes.

## 5 Conclusion

In this study, the drought resistance of 42 lettuce genotypes was assessed under both drought-stressed and normal-irrigated conditions. Thirteen indexes related to morphology, physiology, biochemical characteristics, and osmoregulation were evaluated to determine the genotypes’ drought tolerance. Various analytical techniques, including PCA, membership function value analysis, multiple regression analysis, CDC, D value, and WDC analysis, and cluster analysis, were employed to effectively evaluate the drought tolerance of pakchoi varieties. The assessment of lettuce drought tolerance involved analyzing six indicators through stepwise regression based on the D value: RSA, RV, BDW, SS, SP, and RWC. A digital model was developed to evaluate the drought tolerance of lettuce, and representative varieties were selected from five groups to assess their water resistance ability. The tolerance levels of each variety were validated through measurements of stomatal conductance features, ROS, MDA levels, and antioxidant enzyme activity. This research contributes to the identification of drought-resistant lettuce genotypes and provides a theoretical basis for further investigations into the underlying mechanisms of lettuce drought resistance.

## Data availability statement

The original contributions presented in the study are included in the article/**Supplementary Material**. Further inquiries can be directed to the corresponding author.

## Author contributions

JL, HG and SH designed the experiments. JL and KA drafted the initial manuscript. LW and WW conducted the experiments. BD and HX analyzed the data. GL, BG and XW revised the manuscript. All authors contributed to the manuscript and approved the final manuscript.
