# Supplementary material for: Drought resistance index screening and evaluation of lettuce under water deficit conditions on the basis of morphological and physiological differences
Source: Front Plant Sci. 2023 Sep 15;14:1228084. doi: 10.3389/fpls.2023.1228084 (PMC10540308; doi:10.3389/fpls.2023.1228084)
Supplement: Supplementary file 1 [file Table_1.docx]

Supplementary Material

Drought resistance index screening and varietal evaluation of lettuce under water deficit conditions on the basis of morphological and physiological differences

**Jingrui Li, Kumail Abbas, Lin Wang, Binbin Gong, Shenglin Hou, Weihong Wang, Bowen Dai, Hui Xia, Xiaolei Wu, Guiyun Lü, Hongbo Gao***

*** Correspondence:** Corresponding Author: hongbogao@hebau.edu.cn

**Table S1.** Physical properties of the soil used in the study

| Property | Value |
| --- | --- |
| Clay (%) | 1.85 |
| Silt (%) | 34.42 |
| Sand (%) | 63.73 |
| Field capacity (cm^3^/cm^3^) | 0.34 |
| Soil bulk density (g/cm³) | 1.44 |
