# Supplementary material for: Drought resistance index screening and evaluation of lettuce under water deficit conditions on the basis of morphological and physiological differences
Source: Front Plant Sci. 2023 Sep 15;14:1228084. doi: 10.3389/fpls.2023.1228084 (PMC10540308; doi:10.3389/fpls.2023.1228084)
Supplement: Supplementary file 2 [file Table_2.docx]

Supplementary Material

Drought resistance index screening and varietal evaluation of lettuce under water deficit conditions on the basis of morphological and physiological differences

**Jingrui Li, Kumail Abbas, Lin Wang, Binbin Gong, Shenglin Hou, Weihong Wang, Bowen Dai, Hui Xia, Xiaolei Wu, Guiyun Lü, Hongbo Gao***

*** Correspondence:** Corresponding Author: hongbogao@hebau.edu.cn

**Table S2.** The index values of 42 lettuce cultivars under normal irrigation and water dedicit condition

| Varieties | LN (No.) | | RL (cm) | | RSA (cm^2^) | | | RV (cm^3^) | | | ARD (mm) | | | AFW (g) | | BFW (g) | | | | ADW (g) | | BDW (g) | | SS (mg/g) | | SP (mg/g) | | REL (%) | | | RWC (%) | | |
| --- | --- | --- | --- | --- | --- | --- | --- | --- | --- | --- | --- | --- | --- | --- | --- | --- | --- | --- | --- | --- | --- | --- | --- | --- | --- | --- | --- | --- | --- | --- | --- | --- | --- |
|  | Control | Treatment | Control | Treatment | | Control | Treatment | | Control | Treatment | | Control | Treatment | Control | Treatment | | Control | Treatment | Control | | Treatment | Control | Treatment | Control | Treatment | Control | Treatment | Control | Treatment | | Control | | Treatment |
| 1902 | 26.67 | 22.67 | 81.34 | 55.10 | | 11.58 | 7.56 | | 0.82 | 0.47 | | 0.55 | 0.47 | 301.67 | 198.33 | | 9.45 | 5.76 | 11.85 | | 8.09 | 0.90 | 0.57 | 34.09 | 33.29 | 260.89 | 190.15 | 121.36 | | 128.32 | | 97.67 | 86.42 |
| Malvna | 33.33 | 23.17 | 96.99 | 51.02 | | 6.30 | 2.26 | | 0.12 | 0.04 | | 0.60 | 0.43 | 669.29 | 443.14 | | 11.87 | 6.17 | 12.83 | | 5.82 | 1.18 | 0.43 | 45.89 | 24.02 | 123.93 | 54.15 | 106.01 | | 106.71 | | 84.67 | 68.84 |
| Gelin | 43.25 | 30.00 | 46.27 | 21.92 | | 13.87 | 4.47 | | 0.54 | 0.13 | | 0.79 | 0.56 | 475.67 | 261.15 | | 8.02 | 3.99 | 12.87 | | 5.36 | 0.70 | 0.17 | 40.95 | 16.02 | 301.26 | 123.70 | 113.95 | | 114.58 | | 90.75 | 70.51 |
| Lvdie | 33.00 | 28.50 | 81.34 | 59.72 | | 9.79 | 1.27 | | 0.75 | 0.46 | | 0.51 | 0.47 | 432.00 | 322.50 | | 8.69 | 5.58 | 15.10 | | 10.66 | 0.82 | 0.55 | 21.29 | 46.55 | 107.04 | 173.11 | 130.18 | | 142.22 | | 90.38 | 47.52 |
| ZiXia | 27.83 | 27.00 | 71.30 | 69.28 | | 9.72 | 1.11 | | 0.10 | 0.09 | | 0.54 | 0.53 | 300.00 | 291.67 | | 7.70 | 7.50 | 19.03 | | 18.30 | 0.85 | 0.83 | 21.09 | 41.89 | 288.67 | 92.43 | 120.15 | | 140.20 | | 85.39 | 39.30 |
| Zidie | 26.50 | 25.17 | 159.32 | 147.37 | | 20.74 | 19.00 | | 0.35 | 0.31 | | 0.44 | 0.44 | 384.17 | 362.50 | | 2.12 | 1.93 | 6.24 | | 5.75 | 1.03 | 0.93 | 23.62 | 40.02 | 105.26 | 196.30 | 0.16 | | 0.21 | | 83.73 | 81.67 |
| Sheshou101 | 33.83 | 28.33 | 129.47 | 82.26 | | 8.24 | 5.13 | | 0.55 | 0.29 | | 0.49 | 0.40 | 406.67 | 392.17 | | 9.44 | 5.68 | 13.73 | | 9.01 | 0.25 | 0.15 | 26.02 | 23.95 | 88.15 | 60.67 | 108.59 | | 113.98 | | 85.87 | 75.85 |
| Hongshanhu | 32.83 | 28.17 | 116.76 | 79.03 | | 8.99 | 6.06 | | 0.51 | 0.29 | | 0.52 | 0.46 | 170.00 | 117.50 | | 12.91 | 7.92 | 11.54 | | 7.96 | 0.97 | 0.62 | 15.55 | 15.42 | 279.85 | 204.59 | 110.85 | | 117.22 | | 82.93 | 73.59 |
| Ziya | 28.20 | 18.80 | 91.89 | 42.95 | | 10.84 | 2.06 | | 0.74 | 0.01 | | 0.92 | 0.40 | 302.00 | 201.00 | | 8.07 | 3.16 | 17.55 | | 7.20 | 1.38 | 0.28 | 41.29 | 12.89 | 132.89 | 48.30 | 0.24 | | 0.24 | | 90.98 | 66.97 |
| Lvshen | 31.17 | 27.17 | 130.89 | 105.53 | | 10.21 | 1.52 | | 0.17 | 0.12 | | 1.21 | 1.12 | 197.50 | 157.50 | | 4.53 | 3.13 | 11.24 | | 8.35 | 0.62 | 0.43 | 33.35 | 44.95 | 50.95 | 64.07 | 150.00 | | 167.79 | | 87.70 | 36.50 |
| Lvya | 36.50 | 34.67 | 82.30 | 76.08 | | 16.24 | 14.82 | | 0.28 | 0.25 | | 0.49 | 0.49 | 175.00 | 168.33 | | 19.24 | 17.45 | 12.60 | | 11.48 | 0.57 | 0.51 | 16.49 | 25.89 | 66.67 | 118.81 | 120.61 | | 152.09 | | 86.47 | 84.28 |
| Yushanhong | 25.00 | 23.33 | 103.83 | 93.25 | | 17.79 | 16.17 | | 0.30 | 0.26 | | 0.53 | 0.51 | 286.88 | 187.88 | | 7.66 | 6.71 | 12.52 | | 11.26 | 0.64 | 0.54 | 24.55 | 37.22 | 123.63 | 169.78 | 90.16 | | 113.11 | | 85.30 | 82.41 |
| Tehongzhou | 20.57 | 16.83 | 78.37 | 48.87 | | 12.39 | 7.19 | | 0.48 | 0.25 | | 0.58 | 0.48 | 415.00 | 272.50 | | 8.56 | 4.84 | 25.17 | | 15.84 | 0.61 | 0.36 | 45.75 | 41.49 | 96.59 | 66.30 | 125.27 | | 131.16 | | 85.35 | 75.20 |
| Yeluo | 26.14 | 24.14 | 111.33 | 95.53 | | 8.14 | 7.16 | | 0.40 | 0.32 | | 0.52 | 0.50 | 127.50 | 116.67 | | 12.13 | 10.21 | 22.68 | | 19.83 | 1.07 | 0.87 | 28.62 | 38.70 | 135.78 | 162.59 | 111.45 | | 134.67 | | 80.70 | 77.38 |
| Yidali 151 | 40.17 | 40.00 | 96.55 | 94.73 | | 11.47 | 11.41 | | 0.57 | 0.56 | | 0.55 | 0.54 | 416.67 | 385.83 | | 7.39 | 7.28 | 9.51 | | 9.49 | 1.91 | 1.90 | 22.15 | 56.30 | 45.85 | 97.19 | 81.75 | | 132.44 | | 94.48 | 94.40 |
| Jingyanyidali | 25.50 | 23.33 | 103.33 | 86.83 | | 18.19 | 15.77 | | 0.40 | 0.31 | | 0.56 | 0.54 | 325.00 | 292.50 | | 10.91 | 9.16 | 12.22 | | 10.27 | 0.69 | 0.54 | 30.62 | 39.85 | 187.19 | 202.74 | 104.08 | | 122.92 | | 83.67 | 79.89 |
| Puxijin | 22.67 | 22.50 | 59.43 | 57.89 | | 20.70 | 2.51 | | 0.31 | 0.29 | | 0.51 | 0.51 | 270.50 | 266.50 | | 14.80 | 14.52 | 14.33 | | 14.15 | 0.62 | 0.62 | 19.62 | 9.75 | 127.56 | 54.62 | 119.69 | | 164.43 | | 84.89 | 31.40 |
| Musi | 23.83 | 18.83 | 134.09 | 77.94 | | 42.95 | 8.07 | | 1.24 | 0.59 | | 0.91 | 0.69 | 256.33 | 166.75 | | 11.82 | 6.49 | 6.83 | | 3.50 | 1.22 | 0.69 | 32.02 | 35.75 | 79.69 | 88.42 | 126.65 | | 129.87 | | 87.17 | 51.36 |
| Lvluoma | 34.14 | 31.83 | 85.67 | 76.11 | | 4.86 | 4.38 | | 0.76 | 0.64 | | 0.60 | 0.58 | 392.14 | 201.43 | | 7.42 | 6.48 | 17.95 | | 16.00 | 0.59 | 0.50 | 25.82 | 38.09 | 131.85 | 171.11 | 102.36 | | 127.02 | | 84.87 | 81.79 |
| Ziqueshe | 67.00 | 57.75 | 135.96 | 98.51 | | 17.34 | 11.92 | | 0.40 | 0.23 | | 0.59 | 0.53 | 259.25 | 188.50 | | 11.60 | 7.21 | 15.55 | | 10.85 | 0.74 | 0.49 | 42.29 | 42.22 | 112.89 | 92.89 | 114.99 | | 124.17 | | 89.19 | 79.33 |
| Kana | 27.75 | 22.17 | 78.32 | 48.75 | | 12.37 | 7.15 | | 0.33 | 0.16 | | 0.63 | 0.50 | 288.40 | 271.50 | | 8.23 | 4.62 | 9.43 | | 5.73 | 1.32 | 0.76 | 40.69 | 36.42 | 119.85 | 80.74 | 116.76 | | 121.39 | | 84.08 | 74.08 |
| Jinsen | 20.33 | 18.33 | 104.23 | 87.25 | | 20.42 | 17.16 | | 0.36 | 0.28 | | 0.60 | 0.56 | 498.83 | 432.50 | | 5.27 | 4.28 | 8.75 | | 7.14 | 0.79 | 0.60 | 15.55 | 19.55 | 120.89 | 123.70 | 0.26 | | 0.30 | | 85.47 | 80.91 |
| Yanzhi | 22.29 | 17.57 | 100.76 | 58.47 | | 34.56 | 16.37 | | 0.44 | 0.17 | | 0.80 | 0.60 | 340.13 | 205.83 | | 2.91 | 1.60 | 8.51 | | 4.36 | 0.78 | 0.44 | 39.15 | 29.95 | 140.00 | 84.81 | 105.69 | | 108.35 | | 89.55 | 77.72 |
| Cuiju | 45.00 | 41.33 | 93.61 | 79.48 | | 14.34 | 12.61 | | 0.67 | 0.53 | | 0.54 | 0.52 | 170.00 | 156.68 | | 5.14 | 4.33 | 11.04 | | 9.41 | 0.31 | 0.24 | 27.95 | 37.69 | 136.89 | 151.78 | 109.01 | | 129.83 | | 88.24 | 84.56 |
| Baoshihong | 29.33 | 27.83 | 100.42 | 92.10 | | 14.52 | 13.22 | | 0.17 | 0.15 | | 0.55 | 0.53 | 426.42 | 395.00 | | 10.06 | 9.08 | 8.01 | | 7.26 | 0.50 | 0.43 | 19.49 | 30.02 | 68.00 | 118.67 | 89.18 | | 112.12 | | 81.95 | 79.20 |
| Baoshilv | 24.67 | 23.83 | 62.13 | 58.32 | | 11.65 | 10.90 | | 0.12 | 0.11 | | 0.51 | 0.50 | 502.42 | 453.33 | | 7.86 | 7.37 | 17.51 | | 16.69 | 0.75 | 0.72 | 15.09 | 29.69 | 59.63 | 121.04 | 108.36 | | 142.53 | | 87.86 | 86.43 |
| Xiangyehong | 25.00 | 21.67 | 56.79 | 44.74 | | 8.82 | 6.84 | | 1.81 | 1.18 | | 0.66 | 0.62 | 321.30 | 246.25 | | 14.30 | 9.72 | 13.67 | | 10.06 | 0.57 | 0.39 | 28.75 | 32.09 | 72.67 | 66.52 | 129.47 | | 143.41 | | 88.72 | 82.02 |
| Xiangyelv | 49.50 | 44.00 | 55.08 | 45.08 | | 22.17 | 17.99 | | 0.54 | 0.39 | | 0.47 | 0.44 | 290.83 | 248.33 | | 10.26 | 7.89 | 18.92 | | 15.27 | 0.72 | 0.54 | 33.22 | 40.35 | 198.44 | 196.44 | 107.78 | | 125.44 | | 90.93 | 84.77 |
| Daluoma | 25.33 | 23.00 | 112.83 | 94.54 | | 5.79 | 4.87 | | 0.46 | 0.35 | | 0.64 | 0.60 | 162.50 | 72.50 | | 8.88 | 7.40 | 18.58 | | 15.22 | 1.30 | 0.99 | 32.29 | 41.02 | 116.89 | 124.67 | 121.63 | | 143.01 | | 99.07 | 94.37 |
| Luoshahong | 26.14 | 22.57 | 139.23 | 102.06 | | 18.27 | 12.65 | | 0.28 | 0.17 | | 0.61 | 0.55 | 652.43 | 477.41 | | 19.24 | 12.18 | 5.45 | | 3.82 | 0.94 | 0.63 | 29.35 | 29.49 | 241.78 | 199.26 | 99.96 | | 108.63 | | 82.83 | 73.75 |
| Luoshalv | 41.83 | 36.67 | 193.43 | 156.13 | | 9.38 | 7.49 | | 0.79 | 0.58 | | 0.55 | 0.51 | 392.50 | 317.50 | | 10.31 | 7.21 | 8.07 | | 6.11 | 0.29 | 0.21 | 35.95 | 40.88 | 109.11 | 102.15 | 119.07 | | 137.35 | | 84.73 | 78.90 |
| Yidalishengcai | 31.33 | 24.33 | 62.53 | 35.79 | | 18.14 | 8.06 | | 0.53 | 0.18 | | 1.09 | 0.81 | 625.00 | 373.83 | | 7.65 | 4.13 | 23.04 | | 10.82 | 0.53 | 0.28 | 29.15 | 20.52 | 213.63 | 121.26 | 111.60 | | 113.71 | | 89.63 | 76.10 |
| Ruiluo | 31.17 | 26.33 | 149.35 | 97.93 | | 25.80 | 16.67 | | 0.19 | 0.11 | | 0.58 | 0.50 | 286.67 | 285.75 | | 3.46 | 2.10 | 7.53 | | 5.07 | 0.91 | 0.57 | 33.15 | 30.69 | 86.00 | 60.52 | 95.40 | | 100.37 | | 87.80 | 77.63 |
| Naiyoushengcai | 33.83 | 30.17 | 32.06 | 26.37 | | 7.84 | 6.52 | | 0.59 | 0.44 | | 0.54 | 0.51 | 486.75 | 417.46 | | 9.82 | 7.68 | 12.77 | | 10.36 | 0.72 | 0.54 | 32.55 | 39.95 | 127.33 | 129.70 | 85.94 | | 100.24 | | 84.91 | 79.91 |
| Wojulvsha | 22.17 | 16.00 | 98.23 | 52.20 | | 14.75 | 6.47 | | 1.41 | 0.45 | | 0.56 | 0.41 | 156.21 | 89.17 | | 4.89 | 2.58 | 12.85 | | 6.03 | 0.23 | 0.11 | 43.42 | 25.29 | 243.70 | 136.59 | 110.93 | | 112.72 | | 90.05 | 74.04 |
| Wojuyadan | 28.33 | 26.17 | 100.84 | 87.48 | | 17.61 | 2.01 | | 0.66 | 0.55 | | 0.48 | 0.47 | 196.75 | 180.83 | | 7.70 | 6.59 | 8.03 | | 7.07 | 0.90 | 0.73 | 28.02 | 16.02 | 83.15 | 145.26 | 84.23 | | 102.11 | | 98.78 | 61.68 |
| Lvbei | 23.00 | 22.50 | 127.86 | 124.45 | | 9.50 | 9.20 | | 0.46 | 0.42 | | 0.60 | 0.60 | 157.50 | 153.33 | | 8.40 | 8.22 | 10.86 | | 10.64 | 0.71 | 0.70 | 41.09 | 91.04 | 120.22 | 251.19 | 97.33 | | 130.04 | | 88.12 | 87.80 |
| Meiguodasusheng | 31.00 | 26.83 | 47.91 | 37.70 | | 24.38 | 18.33 | | 0.31 | 0.19 | | 0.50 | 0.46 | 223.33 | 170.83 | | 8.18 | 5.55 | 12.61 | | 9.11 | 2.33 | 1.59 | 46.55 | 49.35 | 149.41 | 133.85 | 96.25 | | 106.13 | | 86.96 | 79.29 |
| Wojunisi | 39.75 | 31.50 | 167.77 | 102.30 | | 16.33 | 8.59 | | 0.55 | 0.27 | | 0.65 | 0.51 | 350.00 | 229.17 | | 5.51 | 3.06 | 15.28 | | 8.25 | 0.29 | 0.17 | 37.22 | 32.96 | 113.56 | 75.70 | 118.34 | | 121.64 | | 89.81 | 78.47 |
| Lvhudie | 35.00 | 31.00 | 59.44 | 48.39 | | 17.49 | 14.04 | | 0.24 | 0.18 | | 0.53 | 0.49 | 375.00 | 311.50 | | 11.16 | 8.57 | 9.05 | | 7.27 | 1.41 | 1.00 | 17.82 | 21.49 | 124.59 | 122.74 | 97.93 | | 113.88 | | 82.81 | 77.15 |
| Lvmeigui | 60.83 | 58.00 | 73.03 | 68.01 | | 12.58 | 11.66 | | 0.50 | 0.45 | | 0.57 | 0.56 | 178.33 | 160.00 | | 19.07 | 17.79 | 5.01 | | 4.71 | 0.98 | 0.93 | 17.89 | 33.95 | 81.56 | 152.67 | 99.91 | | 130.53 | | 86.83 | 84.96 |
| Lvshanhu | 37.00 | 32.00 | 49.62 | 38.07 | | 68.01 | 32.09 | | 0.28 | 0.18 | | 0.61 | 0.56 | 242.85 | 182.75 | | 6.85 | 4.62 | 12.22 | | 8.73 | 0.36 | 0.25 | 27.69 | 33.35 | 186.44 | 34.49 | 105.40 | | 115.52 | | 86.62 | 48.84 |
| Average | 32.11 | 28.00 | 96.71 | 73.94 | | 16.31 | 9.78 | | 0.51 | 0.32 | | 0.61 | 0.54 | 330.65 | 257.76 | | 9.27 | 6.85 | 12.82 | | 9.48 | 0.83 | 0.59 | 30.07 | 33.95 | 137.15 | 122.99 | 101.64 | | 116.25 | | 87.57 | 73.98 |

LN: leaf number; RL: root length; RSA: root surface area; RV: root volume; ARD: average root diameter; AFW: aboveground fresh weight; BFW: belowground fresh weight; ADW: aboveground dry weight; BDW: belowground dry weight; SS: soluble sugar; SP: soluble protein; REL: relative electrolytic leakage; RWC: leaf relative water content. DC: drought resistance coefficient; CDC: comprehensive drought resistance coefficient; D value: drought resistance comprehensive evaluation value; WDC: weight drought resistance coefficient
