# Supplementary material for: Drought resistance index screening and evaluation of lettuce under water deficit conditions on the basis of morphological and physiological differences
Source: Front Plant Sci. 2023 Sep 15;14:1228084. doi: 10.3389/fpls.2023.1228084 (PMC10540308; doi:10.3389/fpls.2023.1228084)
Supplement: Supplementary file 3 [file Table_3.docx]

Supplementary Material

Drought resistance index screening and evaluation of lettuce under water deficit conditions on the basis of morphological and physiological differences

Jingrui Li, Kumail Abbas, Lin Wang, Binbin Gong , Shenglin Hou, Weihong Wang, Bowen Dai, Hui Xia, Xiaolei Wu, Guiyun Lü, Hongbo Gao*

*** Correspondence:** Corresponding Author: hongbogao@hebau.edu.cn

**Table S3.** Analysis of the mean values of all indexes under normal irrigation and water deficit conditions

| Indexes | Treatment | Average value | Coefficient of variation | Standard error | T-value | P-value | Correlation coefficient |
| --- | --- | --- | --- | --- | --- | --- | --- |
| LN | Control | 32.11 | 0.31 | 4.11 | 9.37 | 0.00 | 0.96 |
|  | Treatment | 28.00 | 0.33 |  |  |  |  |
| RL | Control | 96.71 | 0.37 | 22.77 | 8.60 | 0.00 | 0.88 |
|  | Treatment | 73.94 | 0.41 |  |  |  |  |
| RSA | Control | 33.74 | 1.21 | 19.62 | 3.52 | 0.00 | 0.47 |
|  | Treatment | 14.12 | 1.14 |  |  |  |  |
| RV | Control | 0.51 | 0.66 | 0.19 | 5.94 | 0.00 | 0.81 |
|  | Treatment | 0.32 | 0.67 |  |  |  |  |
| ARD | Control | 0.61 | 0.26 | 0.08 | 5.02 | 0.00 | 0.79 |
|  | Treatment | 0.54 | 0.22 |  |  |  |  |
| AFW | Control | 330.65 | 0.41 | 72.79 | 7.33 | 0.00 | 0.89 |
|  | Treatment | 257.76 | 0.42 |  |  |  |  |
| BFW | Control | 9.27 | 0.43 | 2.42 | 9.12 | 0.00 | 0.90 |
|  | Treatment | 6.85 | 0.53 |  |  |  |  |
| ADW | Control | 12.82 | 0.37 | 3.34 | 7.64 | 0.00 | 0.81 |
|  | Treatment | 9.48 | 0.43 |  |  |  |  |
| BDW | Control | 0.83 | 0.51 | 0.24 | 6.84 | 0.00 | 0.84 |
|  | Treatment | 0.59 | 0.60 |  |  |  |  |
| SS | Control | 30.07 | 0.31 | -3.88 | -1.72 | 0.13 | 0.24 |
|  | Treatment | 33.95 | 0.41 |  |  |  |  |
| SP | Control | 137.15 | 0.48 | 14.16 | 1.32 | 0.03 | 0.33 |
|  | Treatment | 122.99 | 0.42 |  |  |  |  |
| REL | Control | 101.64 | 0.31 | -14.61 | -7.64 | 0.00 | 0.94 |
|  | Treatment | 116.25 | 0.31 |  |  |  |  |
| RWC | Control | 99.88 | 0.32 | 20.20 | 4.10 | 0.42 | 0.13 |
|  | Treatment | 79.68 | 0.07 |  |  |  |  |

LN: leaf number; RL: root length; RSA: root surface area; RV: root volume; ARD: average root diameter; AFW: aboveground fresh weight; BFW: belowground fresh weight; ADW: aboveground dry weight; BDW: belowground dry weight; SS: soluble sugar; SP: soluble protein; REL: relative electrolytic leakage; RWC: leaf relative water content.
