# Supplementary material for: Drought resistance index screening and evaluation of lettuce under water deficit conditions on the basis of morphological and physiological differences
Source: Front Plant Sci. 2023 Sep 15;14:1228084. doi: 10.3389/fpls.2023.1228084 (PMC10540308; doi:10.3389/fpls.2023.1228084)
Supplement: Supplementary file 4 [file Table_4.docx]

Supplementary Material

Drought resistance index screening and evaluation of lettuce under water deficit conditions on the basis of morphological and physiological differences

**Jingrui Li, Kumail Abbas, Lin Wang, Binbin Gong, Shenglin Hou, Weihong Wang, Bowen Dai, Hui Xia, Xiaolei Wu, Guiyun Lü, Hongbo Gao***

*** Correspondence:** Corresponding Author: hongbogao@hebau.edu.cn

**Table S4.** Classification of comprehensive evaluation indexes of lettuce genotypes under water deficit conditons

| Indexes | Membership function | | | | |
| --- | --- | --- | --- | --- | --- |
|  | Ⅰ | Ⅱ | Ⅲ | Ⅳ | Ⅴ |
| LN | 1.00 | 0.96 | 0.91 | 0.84 | 0.73 |
| RL | 0.98 | 0.93 | 0.85 | 0.69 | 0.53 |
| RSA | 1.00 | 0.93 | 0.70 | 0.53 | 0.35 |
| RV | 0.98 | 0.89 | 0.78 | 0.57 | 0.30 |
| ARD | 0.99 | 0.98 | 0.95 | 0.87 | 0.69 |
| AFW | 0.93 | 0.93 | 0.81 | 0.78 | 0.61 |
| BFW | 0.99 | 0.93 | 0.82 | 0.61 | 0.51 |
| ADW | 1.00 | 0.94 | 0.84 | 0.67 | 0.46 |
| BDW | 1.00 | 0.93 | 0.79 | 0.63 | 0.43 |
| SS | 2.54 | 1.81 | 1.22 | 1.10 | 0.63 |
| SP | 2.12 | 1.90 | 1.02 | 0.80 | 0.58 |
| REL | 1.62 | 1.29 | 1.19 | 1.07 | 1.01 |
| RWC | 1.00 | 0.98 | 0.86 | 0.79 | 0.78 |
| CDC | 0.98 | 0.83 | 0.64 | 0.46 | 0.28 |
| D-value | 1.24 | 1.11 | 0.90 | 0.77 | 0.59 |
| WDC | 1.63 | 1.48 | 1.21 | 1.01 | 0.76 |

Ⅰ, Ⅱ, Ⅲ, Ⅳ, and Ⅴ represent different drought resistance levels. LN: leaf number; RL: root length; RSA: root surface area; RV: root volume; ARD: average root diameter; AFW: aboveground fresh weight; BFW: belowground fresh weight; ADW: aboveground dry weight; BDW: belowground dry weight; SS: soluble sugar; SP: soluble protein; REL: relative electrolytic leakage; RWC: leaf relative water content. CDC: comprehensive drought resistance coefficient; D value: drought resistance comprehensive evaluation value; WDC: weight drought resistance coefficient.
